# BMV and CCMV-Based Viral Nanoparticles for Delivery of *N*‑Desmethyl-Tamoxifen as Treatment of Triple-Negative Breast Cancer

**DOI:** 10.1021/acsomega.5c11566

**Published:** 2026-02-27

**Authors:** Elizabeth Loredo-García, Pierrick G. J. Fournier, M. Mariana Herrera-Hernandez, Kanchan Chauhan, Ana G. Rodriguez-Hernandez, Rafael Vazquez-Duhalt, Ruben D. Cadena-Nava

**Affiliations:** † 87793 Centro de Nanociencias y Nanotecnología, Universidad Nacional Autónoma de México (UNAM), Km. 107 Carretera Tijuana-Ensenada, Ensenada, Baja California CP. 22800, México; ‡ 7082 Centro de Investigación Científica y de Educación Superior de Ensenada, Baja California, (CICESE), Carretera Ensenada-Tijuana 3918, Ensenada, Baja California CP. 22860, México

## Abstract

Viral nanoparticles (VNPs) based on BMV (Brome mosaic virus) and CCMV (Cowpea chlorotic mottle virus) were developed for targeted delivery of *N*-desmethyl-tamoxifen (NDMT), an active metabolite of tamoxifen with potent antiestrogenic activity. *In silico* simulations predicted that BMV could load 20% more NDMT than CCMV, which was experimentally confirmed by fluorescence assays and physicochemical characterization. VNPs showed efficient cell internalization in triple-negative breast cancer cells (4T1), localizing both in the cytoplasm and the nucleus, where NDMT exerts its therapeutic action. Cell viability assays revealed that BMV-NDMT and CCMV-NDMT are significantly more effective than the free NDMT, showing lower IC_50_ in both cell lines. Furthermore, in a 4T1 murine breast cancer model, BMV-NDMT reduced tumor volume by 44% and lung metastasis by 74%, demonstrating superior antitumor and antimetastatic activities compared to controls. These results highlight the potential of VNPs from plant viruses as efficient and biocompatible delivery systems for breast cancer treatment, with significantly lower doses than free drug.

## Graphical abstract

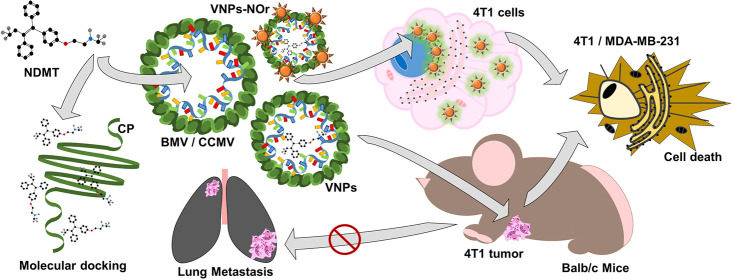

## Introduction

Breast cancer is one of the leading causes of death worldwide, with 2.3 million incident cases and approximately 665,600 deaths recorded in 2022. It is projected that by 2050, incidence will increase by 43% and mortality by 68%, highlighting the urgent need to develop more effective and safer therapeutic strategies.^1^ Breast cancer is classified by hormone receptor (HR) and human epidermal growth factor receptor 2 (ERBB2) expression into three main subtypes. Hormone receptor-positive (HR^+^/ERBB2^–^) and triple-positive (HR^+^/ERBB2^+^) tumors represent 70% of cases and are treated with endocrine therapy, often combined with chemotherapy. In contrast, triple-negative tumors (HR^–^/ERBB2^–^) lack these target receptors, and are treated primarily with surgery and chemotherapy.^2^

Tamoxifen, a selective estrogen receptor modulator, is a prodrug converted into active forms *via* cytochrome P450-mediated hepatic metabolism, particularly by the CYP2D6 enzyme.^3^ Genetic alterations or variability in CYP2D6 can reduce the production of active metabolites such as *N*-desmethyl-tamoxifen (NDMT), 4-hydroxy-tamoxifen (4HT), and endoxifen (ENDO), which are 30- to 100-fold more antiestrogenic than tamoxifen.^4^ NDMT, the predominant metabolite in the patient’s serum, is primarily produced (92%) by the enzymes CYP3A4, CYP3A5, and CYP2C19. NDMT is then further transformed by CYP2D6 into ENDO, while only 7% of tamoxifen is converted to 4HT by the enzyme CYP2D6.^5,6^ Although ENDO is a more potent blocker of ERα in ER-positive cells, both NDMT and ENDO also inhibit aromatase in HR-negative cells.^7^ NDMT was selected for this proof-of-concept study due to its long serum half-life and clinical data showing that in tamoxifen-treated patients, the plasma concentration of NDMT is more than 70 times higher than that of 4HT and 11 times higher than that of ENDO, making it an ideal candidate to validate the loading and delivery capability of our viral nanoparticle platform (VNP).^8–10^ Despite its potent antiestrogenic activity, NDMT is highly hydrophobic, which limits its solubility, bioavailability, and therapeutic efficacy. The delivery of hydrophobic drugs remains a persistent challenge in developing cancer therapy. Advances in nanotechnology, however, have enabled the design of nanoparticle-based delivery systems that can overcome these limitations. Among these, virus-like nanoparticles (VLPs) can efficiently internalize into cells and release their cargo while preserving drug activity.^11^ Previous studies have explored the use of nanocarriers, such as carbon nanotubes and gold nanoparticles conjugated to NDMT, which have enhanced its *in vitro* efficacy.^12,13^ However, these systems present challenges in terms of biocompatibility and scalability.

VLPs and VNPs offer distinct advantages and challenges. Although VLPs can achieve high encapsulation efficiency for hydrophobic small molecules, weak or nonspecific interactions within the capsid can lead to drug leakage. Additionally, VLP structural stability is highly dependent on pH, temperature, and ionic strength, often requiring protein engineering to maintain integrity and cargo retention. In contrast, VNPs derived from whole viruses (*e.g.*, plant or bacteriophage-based systems) retain their genomic RNA, which acts as a scaffold to stabilize the nucleoprotein complex. This native architecture often confers greater robustness to changes in pH, temperature, and ionic strength compared to empty VLPs, as well as a higher intrinsic drug-loading capacity.^11–15^ Their noninfectious nature in mammals also minimizes biosafety concerns. Potential immunological responses from repetitive dosing could be addressed by surface functionalization strategies such as PEGylation.^14^ The VNP approach has been studied mainly with filamentous and tubular virus structures like tobacco mosaic virus (TMV) and potato virus X (PVX), where RNA serves as a structural template that determines the length of the nucleoprotein complex. As a result, TMV-based VNPs show uniform dimensions (300 × 18 nm^2^), unlike their VLP counterparts, which exhibit variability in length.^15^ On the other hand, quasi-spherical viruses such as bromide mosaic virus (BMV) and cowpea mosaic virus (CCMV), both approximately 28 nm in diameter, have been widely used to encapsulate different molecules and materials, including inorganic nanoparticles, nucleic acids, and bioactive molecules. Furthermore, recent studies have demonstrated that these virus possess the ability to internalize into mammalian cells. Based on previous research conducted with cowpea mosaic virus (CPMV), it has been proposed that CCMV internalization could be mediated by specific interactions between the viral capsid and cell surface proteins, such as vimentin, reinforcing the potential of these viral nanoparticles (VNPs) as nanocarriers in biomedical applications.^16–19^ Several reports demonstrate the potential of these VNPs for chemotherapeutic drug delivery in cancer therapy.^20–22^

Therefore, the objective of this study was to develop a potent therapeutic strategy for triple negative breast cancer (TNBC) by leveraging the HR-independent cytotoxic effects of NDMT while overcoming its pronounced hydrophobicity using VNPs. Two structurally similar but physicochemically distinct plant viruses, BMV and CCMV, were compared to identify the most effective delivery platform. A rational workflow was employed: first, *in silico* docking was used to predict drug loading; their *in vitro* efficacy was evaluated, and finally, the antitumor and antimetastatic potential of the lead candidate was assessed *in vivo*. This approach provides both novel nanotherapeutic and insights into structural features of VNPs that govern their functionality as drug carriers.

## Results and Discussion

### Theoretical Quantification of *N*-Desmethyl-Tamoxifen

Protein–ligand interaction plays an important role in controlled drug delivery using self-assembling nanocarriers. Identifying key interactions for charge balance, the shape, charge, and hydrogen bonds between the drug and the protein docking sites is essential and must be considered.^23^ The theoretical number of NDMT molecules that can dock to BMV and CCMV capsid proteins was determined by molecular docking simulations using Autodock Vina. Figure 1 illustrates some interactions between the drug and the viral capsid proteins. For BMV, 10 NDMT molecules were coupled per protein (Figure 1A), with affinity energies ranging from −5.6 to −4.3 kcal/mol. 85.3% of the interactions were hydrophobic, where the main amino acids involved were alanine (15.7%) and glutamate (12.8%). In addition, the rest of the electrostatic interactions were of the hydrogen bond type (16.7%), these interactions were mediated by the nitrogen and oxygen atoms of NDMT were observed, where the most involved amino acids were alanines and phenylalanines, both with a 16.6% participation.

**Figure 1:**
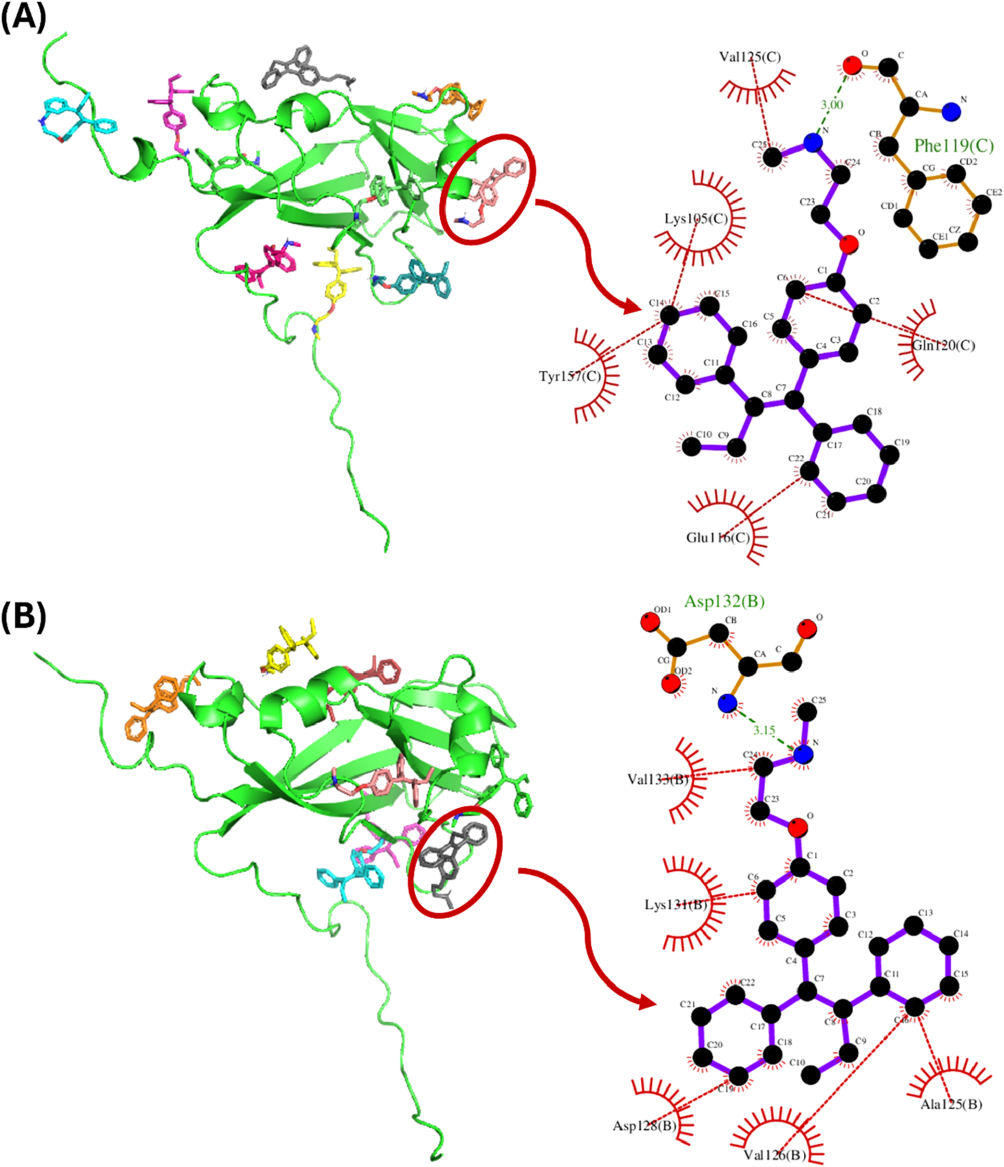
Molecular docking between NDMT and BMV and CCMV proteins: (A) Docking between NDMT and BMV protein, (B) Docking of NDMT and CCMV protein. Hydrophobic interactions (red), hydrogen bonds (green). Molecular docking was performed using Autodock Vina. Images developed in PyMol and LigPlus.

In the case of CCMV, it was estimated that 8 NDMT molecules can be attached per protein (Figure 1B), with affinity energies varying between −5.6 and −4.7 kcal/mol. In this case, 88.5% of the interactions were hydrophobic, with alanines (19.6%) and valines (16.0%) standing out. The remaining 11.5% corresponded to hydrogen bonds, mainly involving leucine and serine, with a 28.0% participation. The binding sites, affinity energies, and types of interaction differed between BMV and CCMV (Tables S1 and S2 in Supporting Information).

The *in silico* docking study of the drug NDMT was performed in both BMV and CCMV proteins. The results indicate that BMV showed more affinity sites (180) with the drug, even though the energetic values of both nanoparticles were similar (−5.3 kcal/mol).

Although BMV and CCMV belong to the same Bromovirus family and share similar characteristics, their protein structures differ by approximately 30%. These structural differences influence the surface charge of the viruses. The isoelectric point of BMV is 5.2, while for CCMV it is 3.7. These variations explain the higher capacity of BMV to load NDMT molecules compared to CCMV.^24^

Although considered weak electrostatic forces, hydrophobic interactions, and hydrogen bonds play a crucial role in biological processes such as protein folding and provide stability to protein complexes.^25,26^ Hydrogen bonds, formed between hydrogen atoms and electronegative elements such as oxygen and nitrogen, are stronger than hydrophobic interactions, which occur when hydrophobic molecules cluster together to reach an energetically stable state.^27,28^ These interaction differences explain the observed variations in drug–protein docking, attributed to the physicochemical and structural differences between BMV and CCMV proteins.

It is important to note that these docking simulations were performed on isolated capsid proteins and do not account for the presence of the viral RNA within the intact virion, which could potentially occupy some interior sites. However, the predominance of hydrophobic interactions predicted for NDMT binding suggests a high affinity for the same types of hydrophobic protein patches that interact with the nitrogenous bases of the RNA. This implies that NDMT loading could occur through competition with RNA bases or by intercalation within the RNA structure itself. The strong agreement between our *in silico* predictions and subsequent experimental loading efficiencies provides support for the functional relevance of these identified interaction sites.

### Characterization of *N*-Desmethyl-Tamoxifen

The successful synthesis and purification of NDMT from tamoxifen was corroborated by mass spectrometry. In the extracted ion chromatogram (EIC) (Figure S1), a peak at 358.21 *m*/*z* is observed, coinciding with the [M + H]^+^ peak, a value also reported in the literature for NDMT.^29^ Further, structural differences between TAM and NDMT were analyzed by Fourier-transform infrared (FTIR) spectroscopy (Figure 2). The appearance of a broad absorption band within the 3300–3500 cm^–1^ region indicates the presence of N–H stretching vibrations, characteristic of secondary amines. The absence of a corresponding band in TAM in this region is consistent with its tertiary amine structure. Additional peaks at 2522.6 cm^–1^ and 2459.45 cm^–1^ corresponding to N–H stretching vibrations were also observed. Notably, both spectra exhibit multiple peaks in the 2800–3000 cm^–1^ and 1500–1600 cm^–1^ range, corresponding to C–H stretching vibrations and C=C stretching vibrations within aromatic rings, respectively. The similarity in this region indicates that the aromatic framework remains largely unchanged postdemethylation. However, slight peak shifts for C–H stretching of aliphatic region ∼2800–2500 cm^–1^ show the change in the chemical environment due to the demethylation process. This comparative analysis revealed the successful demethylation of TAM.

**Figure 2:**
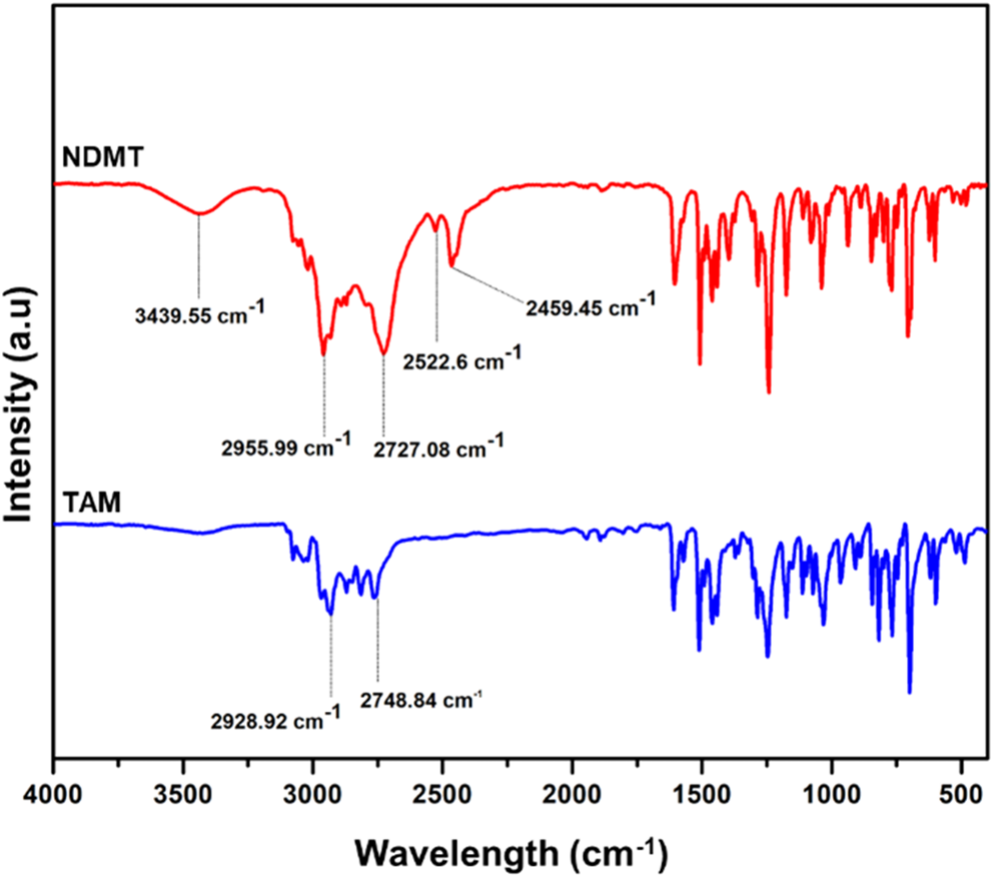
FTIR characterization: Spectra of tamoxifen (TAM) (blue) and *N*-desmethyltamoxifen (NDMT) (red).

In addition, NDMT was characterized by fluorescence spectroscopy. After excitation at 270 nm, the emission spectra of solutions of NDMT at different concentrations were recorded from 350 to 420 nm, and three representative peaks were observed in the spectrum at 365 nm, 385 nm, and one of lower intensity at 405 nm. A fluorescence intensity curve as a function of drug concentration was obtained, which was fitted to a logistic equation model (Figure S2A,B). The logistic equation models the growth of a variable; it has been applied in probability models using logistic regression based on relative fluorescence intensity to distinguish population differences, achieving a specificity of 95.8% in classifying the populations.^30,31^ This analysis allowed the establishment of a quantitative relationship between NDMT concentration and its fluorescence signal, providing a tool for drug quantification.

### Loading of *N*-Desmethyl-Tamoxifen in BMV and CCMV

According to eq 4 described in the Methods section, *in silico* analysis predicted that 200 μg of BMV and CCMV could couple 27.9 μg and 22.3 μg of NDMT, respectively, indicating that BMV has an approximately 20% greater loading capacity than CCMV. Experimentally, an excess of NDMT (30 μg) in PBS buffer was used to ensure complete saturation. The results showed that BMV coupled 11.2% more NDMT than predicted *in silico*, while CCMV coupled 32.1% less (Table 1). The experimental loading rate of NDMT per viral particle was calculated using the concentration determined by fluorescence (Figure S2) according to eq 1, where *m*_f_ represents the mass of NDMT, PM_f_ is the molecular weight of NDMT (357.5 g/mol), where *m*_v_ represents the mass of the virus and *P*_V_ is the weight of the virus (approximately 4.6 × 10^6^ g/mol). These findings indicate that BMV has a higher loading efficiency compared to CCMV.

1 $${\mathrm{NDMT}}_{\mathrm{VNP}}=\frac{m_{f}\times P_{v}}{m_{v}\times {\mathrm{PM}}_{f}}$$

**Table 1:** *N*-Desmethyl-Tamoxifen Coupled to BMV and CCMV Capsids

	number of docked molecules per virion		
nanovehicle	experimentally^a^	*In silico* ^b^	experimentally/*in silico* ^c^
BMV-NDMT	2002 ± 63	1800	111.2%
CCMV-NDMT	978 ± 33	1440	67.9%

a The number of NDMTs that are coupled to BMV and CCMV virions experimentally (*n* = 9 synthesis).

b The number of NDMTs that coupled to BMV and CCMV virions *in silico*.

c Comparison between NDMTs that are coupled to BMV and CCMV virions experimentally *vs* *in silico*.

The morphology and size of NDMT-loaded VNPs were analyzed by transmission electron microscopy (TEM) and dynamic light scattering (DLS). The nanocarriers were successfully synthesized with the two viruses used, BMV and CCMV. TEM micrographs showed that the VNPs maintain the morphology of the viruses after the loading and purification process. The BMV native virus had an average diameter of 27.56 ± 2.1 nm, and CCMV has a diameter of 28.34 ± 2.3 nm (Figure S3). However, TEM analysis showed distinct morphological features after NDMT loading. BMV-NDMT VNPs showed a size increase to 35.7 ± 4.5 nm (Figure 3A,3B), whereas CCMV-NDMT VNPs exhibited a smaller measured diameter of 25.5 ± 2.3 nm (Figure 3D,3E) compared to the native virus. This apparent size reduction for CCMV is likely a TEM artifact that could be caused by alteration in surface adhesion and contrast due to drug conjugation. The particle size in solution, provided by DLS, shows a consistent hydrodynamic diameter increase for both VNPs: 31.5 ± 2.1 nm for BMV-NDMT and 30.8 ± 3.0 nm for CCMV-NDMT (Figure 3C,F). This confirms successful drug incorporation in both systems. The disparity between TEM and DLS data for CCMV-NDMT highlights the different physical information provided by each technique and suggests that NDMT conjugation induces a more pronounced surface modification on CCMV, leading to particle flattening on grids, while reinforcing the BMV structure more uniformly. This size difference highlights how the physical conjugation of identical drugs can result in nanoparticles with different physicochemical properties depending on the biochemical properties of the viral capsids.

**Figure 3:**
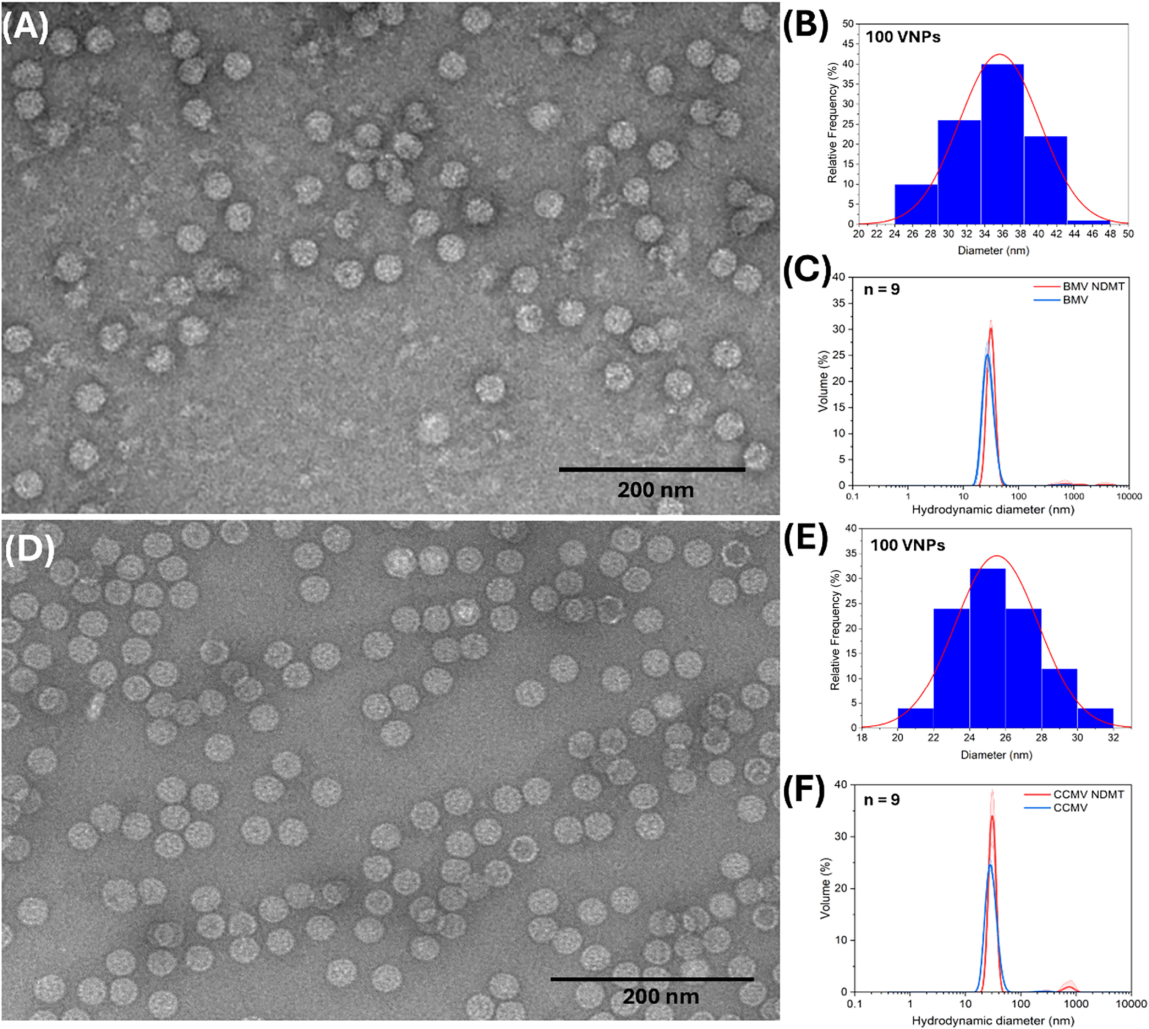
VNPs of BMV and CCMV sizes: (A) TEM micrographs of BMV-NDMT VLPs. (B) Diameter distribution of BMV-NDMT VNPs obtained by TEM images (*n* = 100 VNPs). (C) Hydrodynamic diameter of BMV and BMV-NDMT VLPs obtained by DLS (*n* = 9 measurements). (D) TEM micrographs of CCMV-NDMT VNPs. (E) Diameter distribution of CCMV-NDMT VNPs obtained by TEM images (*n* = 100 VNPs). (F) Hydrodynamic diameter distribution of CCMV and CCMV-NDMT VNPs obtained by DLS (*n* = 9 measurements). TEM micrograph scale corresponds to 200 nm.

Furthermore, fluorescence spectroscopy showed that BMV can load 51.1% more NDMT than CCMV, which is in accordance with *in silico* results but at a higher ratio (Table 1). These findings are consistent with our previous studies. In particular, for BMV and CCMV viruses, pH and molecular interactions do not show significant variations in aqueous solutions containing up to 50% DMSO; furthermore, increasing the pH to 7.4 improves charging conditions, favoring greater coupling of hydrophobic molecules. However, higher solvent concentrations and alkaline aqueous environments compromise the structural integrity of the viral particle.^16,32^ Therefore, while a more hydrophobic environment might initially favor drug solubility, it would likely disrupt the essential protein–protein and protein-RNA interactions that maintain the VNP structure, ultimately decreasing effective loading.

To explain the differences in loading capacity between BMV and CCMV, a pairwise sequence alignment was performed between the C chains of the capsid proteins of both viruses (Figure S4). It was found that 10% of the amino acids involved in drug–protein interactions in BMV belong to regions where the BMV and CCMV proteins are different. In addition, BMV has 180 additional glutamic acid residues compared to CCMV (one more per capsid protein). The deprotonation of these residues under neutral or basic pH conditions, such as in PBS buffer, may promote swelling of BMV VNPs. We propose that this swelling could facilitate a higher NDMT loading. These physicochemical differences could explain the greater capacity of BMV to dock NDMT compared to CCMV. In addition to capsid proteins, we hypothesize that NDMT could also dock at other sites within the virions. Because BMV and CCMV have an internal empty cavity of ∼ 8 nm in diameter,^33^ which could serve as a hosting site for NDMT, this cavity would remove the drug from a hydrophilic environment, thereby improving its stability. Alternatively, NDMT could interact with viral RNA, whose hydrophobic nitrogen bases might facilitate its docking. However, further studies are needed to verify the binding of the drugs to RNA.

### Cellular Internalization of VNPs

The internalization of viral nanoparticles (VNPs) in 4T1 murine breast cancer cells, was assessed using VNPs labeled with the NanoOrange (NOr) fluorophore and analyzed by confocal microscopy. Labeled BMV-NDMT and CCMV-NDMT VNPs showed hydrodynamic diameters of 37.8 ± 5.4 nm and 28.2 ± 5.4 nm, respectively (Figure S5A). Indicating that NOr did not alter the size of VNPs compared to native viruses. The emission spectrum of NOr-labeled VNPs exhibited a peak around 570 nm (Figure S5B).

Figure 4 shows the confocal microscopy images of 4T1 cells exposed to NOr-labeled VNPs. In the images, the cytoskeleton was labeled with rhodamine-phalloidin (green), the cell nucleus with DAPI (blue), and VNPs with NOr (orange). The colocalization of these fluorophores is shown in the D, H, and L boxes of each set of images.

**Figure 4:**
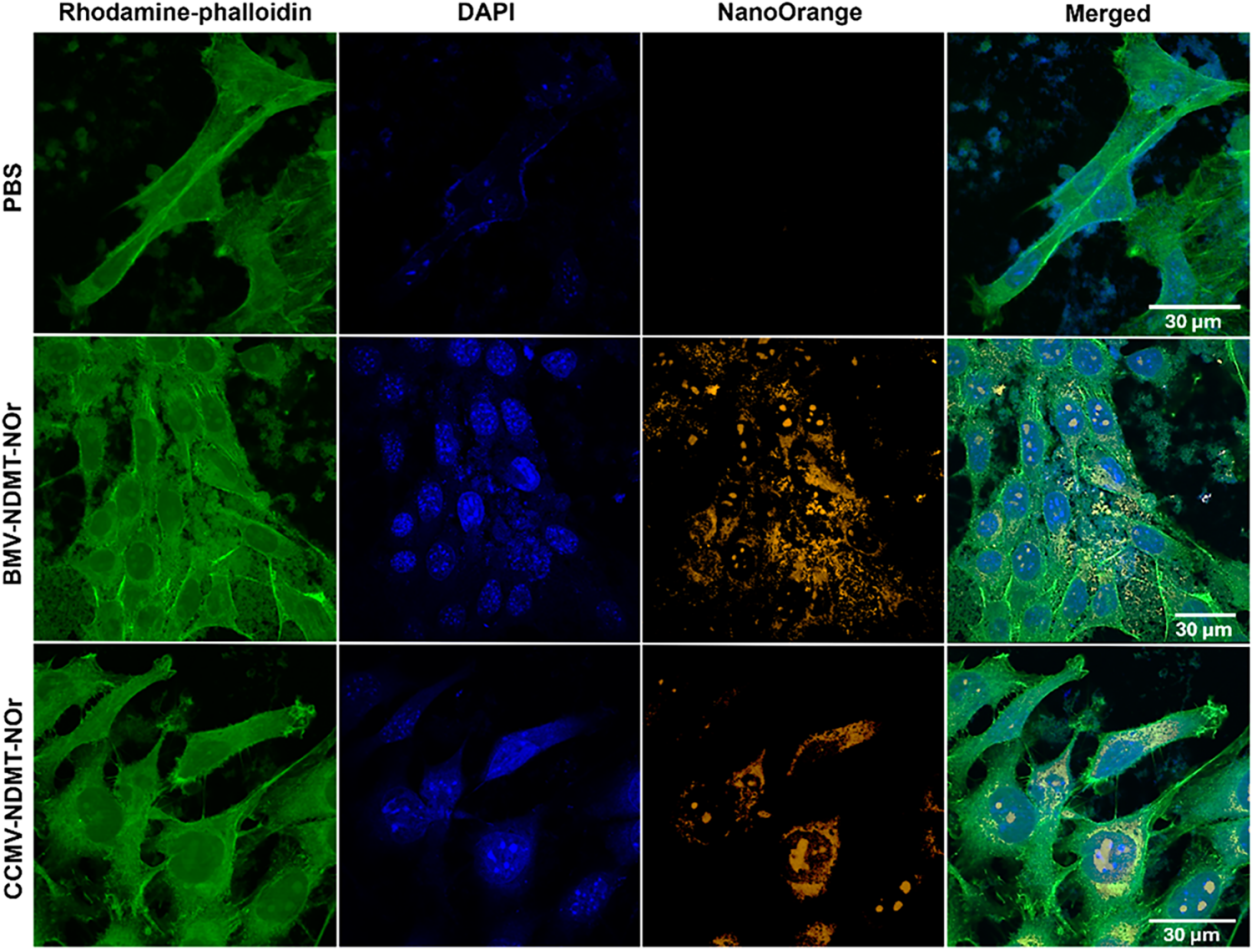
Confocal micrograph of 4T1 cells: The green channel (first column) corresponds to rhodamine-phalloidin, which stains the cytoskeleton. The blue channel (second column) corresponds to DAPI, which stains the nuclei. The orange channel (third column) corresponds to either PBS (negative control), BMV-NDMT-Nor, or CCMV-NDMT-Nor. Co-localization of the three channels is shown in the fourth column. Scale bar: 30 μm.

In the PBS-treated control (Figure 4, first line), no orange signal was observed in the cells, due to the absence of NOr or VNPs labeled with NOr. In contrast, cells treated with BMV-NDMT-NOr and CCMV-NDMT-NOr (Figure 4, second and third lines, respectively) showed orange fluorescence in the cytoplasm and cell nuclei, indicating successful internalization of VNPs. In addition, some agglomerations of VNPs were observed in the extracellular space, suggesting a possible interaction with the extracellular matrix prior to internalization.

These results demonstrate that both BMV-NDMT and CCMV-NDMT are efficiently internalized by 4T1 cells, supporting their potential as drug nanocarriers for NDMT in the treatment of breast cancer.

We previously reported that plant viruses like the BMV and CCMV can be internalized in breast cancer cells.^34^ In addition, BMV and CCMV can be efficiently internalized by endocytosis such similar plant viruses as CPMV (Cowpea mosaic virus).^35^ However, the nuclear internalization of these viral nanoparticles (VNPs) in cells and their localization in both the cytoplasm and the nucleus of 4T1 cells is described for the first time in this study, supported by confocal microscopy. An intense NanoOrange (NOr) signal was observed around the nuclear membrane, the site of action of NDMT, where it blocks estrogen receptors.^11^

The internalization of VNPs into the 4T1 cell line is of particular relevance since mouse models of 4T1 tumors are widely used to study cancer development and progression and to evaluate antitumor therapies. When introduced orthotopically, 4T1 cells can metastasize to the lungs, liver, brain, and bones, closely mimicking the progression of breast cancer in humans.^36^ These findings highlight the importance of VNPs as nanocarriers of NDMT for the treatment of breast cancer.

### Cell Viability

BMV did not compromise MDA-MB-231 cell viability; in fact, an increase of 20% in cell activity was observed. In contrast, CCMV reduced cell viability by approximately 5%. Tumor cells can reprogram their metabolism to support rapid proliferation. Due to their high energy demands, they consume nutrients from their environment and can utilize viral proteins as an energy source. This cell growth phenomenon has been reported with certain viruses such as BMV, CCMV, phage MS2, and the HBx protein of the hepatitis B virus (HBV).^16,37–40^ Furthermore, the persistence of CCMV RNA after a 24-h incubation with murine macrophage cells (RAW 264.7) has been shown to be lower and even undetectable after 72 h, suggesting that the viral genetic material is processed by the cells for disposal or recycling.^41^

For NDMT-loaded VNPs, a concentration-dependent decrease in cell viability was observed (Figure 5A). BMV-NDMT showed a significant effect starting at 4 ng/μL, while CCMV-NDMT was effective starting at 1 ng/μL. Dose–response curve analysis (Figure S6A,B) indicated that the IC_50_ for BMV-NDMT was 4.95 ± 0.42 ng/μL, while for CCMV-NDMT it was 7.8 ± 0.77 ng/μL. Free NDMT reduced cell viability by 50% at a concentration of 12 ng/μL, but in all cases was less effective than loaded VNPs. Between 1 and 8 ng/μL, significant differences were observed between BMV-NDMT and CCMV-NDMT, but at concentrations higher than 10 ng/μL, both systems showed similar effects on MDA-MB-231 cells.

**Figure 5:**
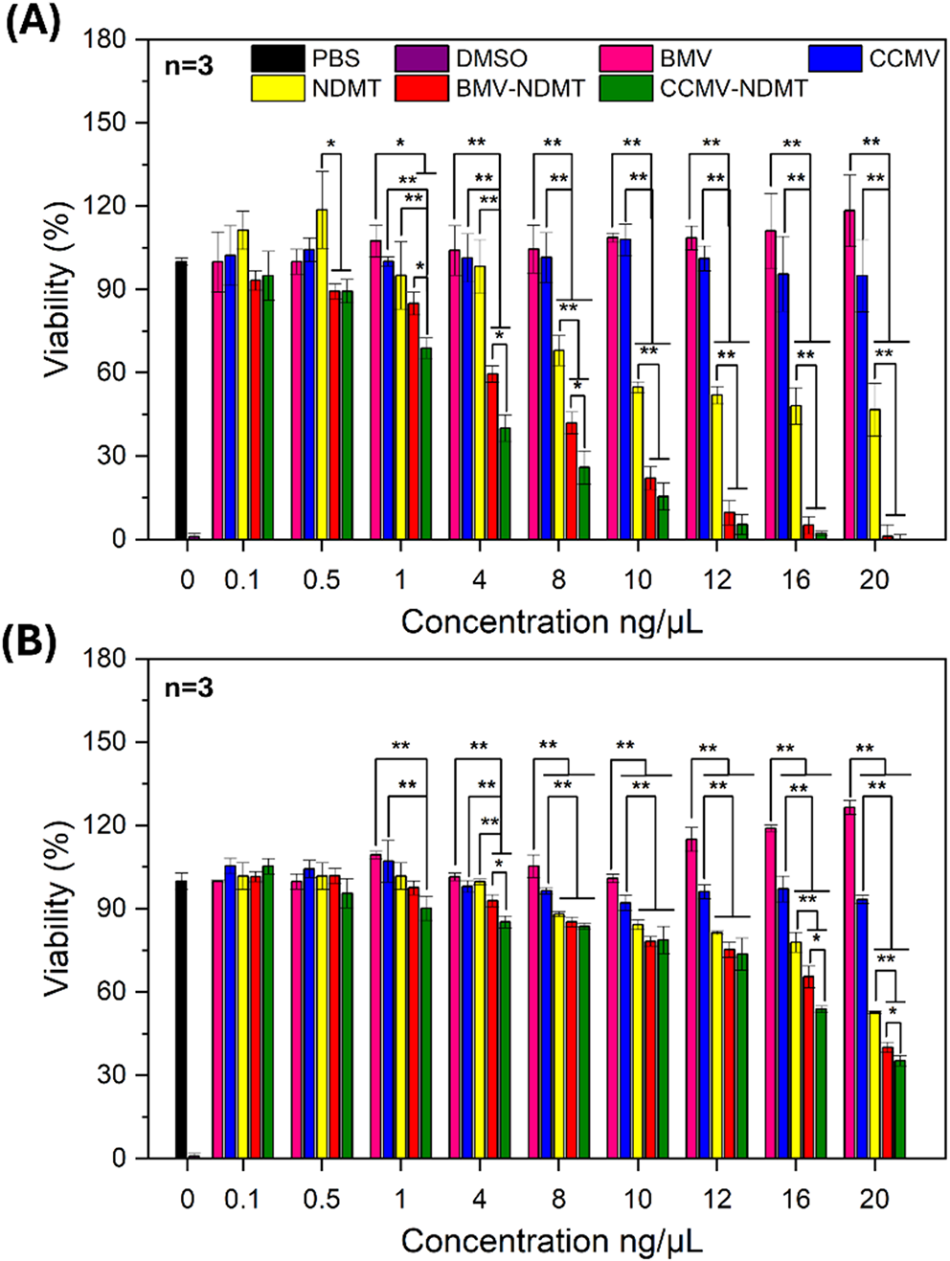
Cell viability of MDA-MB-231 and 4T1: (A) Viability of MDA-MB-231 cells exposed to the treatments (*n* = 3). (B) Viability of 4T1 cells exposed to the treatments (*n* = 3). In the histograms, the results from BMV-NDMT are marked in red, from CCMV-NDMT marked in green, and control experiments, BMV in pink, CCMV in blue, NDMT in yellow, PBS in black, and DMSO marked in violet. The concentration shown in the graph indicates the concentration of the drug loaded in the VNP. The amount of drug and virus present in the VNP determined the concentration of free drug and unloaded virus in the assay. Bars represent the average viability, and error bars represent the standard deviation (SD). Statistical analyses One-way ANOVA, Tukey test, *P < 0.05, **P < 0.005.

In the 4T1 cell line (Figure 5B), BMV did not negatively affect cell viability, while CCMV reduced viability by 10% at 40 ng/μL. BMV-NDMT decreased cell viability starting at 1 ng/μL, with an IC_50_ of 17.37 ± 0.64 ng/μL (Figure S6C,D). Meanwhile, CCMV-NDMT showed a significant effect starting at 4 ng/μL, with an IC_50_ of 16.52 ± 0.50 ng/μL. Free NDMT reduced cell viability by 55% but was less effective than loaded VNPs. At high concentrations (16–20 ng/μL), significant differences were observed between BMV-NDMT and CCMV-NDMT in 4T1 cells.

The BMV did not show cytotoxic effects at the concentrations evaluated, even was evidenced an increase in cell viability of up to 20% in MDA-MB-231 and 30% in 4T1. This phenomenon may be attributed to the high energy demand of cancer cells, which utilize viral proteins as an energy source to meet their bioenergetic needs.^42,43^ Viability assays demonstrated that NDMT-loaded VNPs are more effective than the free drug, with statistically significant differences in IC_50_ between BMV-NDMT and CCMV-NDMT (*P < 0.05). Furthermore, NDMT-loaded VNPs were more active in MDA-MB-231 cells than in 4T1 cells (**P < 0.005, Figure S7).

The significant cytotoxicity of NDMT-loaded VNPs in ER-negative MDA-MB-231 and 4T1 cell lines confirms that the observed antitumor effects are mediated through ER-independent pathways. Although ER-negative cells do not depend on estrogen for survival, tamoxifen and its metabolites can exert potent effects by modulating key survival pathways. For instance, beyond the inhibition of aromatase,^44,45^ tamoxifen induces apoptosis in MDA-MB-231 cells by reducing phosphorylation of Akt at Ser473.^46^ AKT is a critical enzyme that regulates critical processes such as glucose uptake, protein synthesis, and cell proliferation. This rationale is further supported by the high antitumor and antimetastatic activity observed in our *in vivo* 4T1 model, which is unequivocally ER-negative. Previous studies support our findings. NDMT-functionalized nanotubes reduce the cell viability of MDA-MB-231 4-fold more than the free drug, with an IC_50_ of ∼0.04 ng/μL.^12^ On the other hand, NDMT-conjugated gold nanoparticles are 2.7 times more effective than the free drug in MCF-7 cells and exhibited an IC_50_ of ∼0.46 ng/μL.^13^ Our results confirm that NDMT-loaded VNPs are more effective than the free drug and exhibit IC_50_ at similar concentrations to those reported for other nanocarriers, 4.95 ± 0.42 ng/μL (13.84 μM).

The cell viability findings support the hypothesis that VNPs produce better drug internalization, which are in close agreement with the results obtained from the *in vitro* internalization assay. The magnitude of cytotoxicity by VNPs in these cell lines was found to be always more prominent than that of the free drug. The reasons for the improved *in vitro* efficacy could be better drug internalization and release of NDM tamoxifen inside the cells. Although both BMV and CCMV demonstrated efficient cellular internalization as nanocarriers, *in vivo* assays in murine models were performed exclusively with BMV-NDMT. This selection was based on the greater drug-loading capacity observed with BMV, as well as the absence of cytotoxic effects in the MDA-MB-231 and 4T1 cell lines. In contrast, CCMV induced a decrease in cell viability of approximately 5% and 10% in these cell lines, respectively. Furthermore, previous studies have shown that CCMV can activate macrophages, increasing their immunogenicity and, consequently, limiting its applicability in certain therapeutic strategies, including the one evaluated in the present study.^34^

### Mouse Model of Breast Cancer

The 4T1 cells form tumors in BALB/c mice that are visible and palpable on day 9. The weight of mice treated with BMV-NDMT, along with PBS, BMV, and NDMT controls, is shown in Figure S8A. All mice gained weight from day 0 to day 24. However, the PBS, BMV, and NDMT groups showed weight loss between days 24 to 26, while the BMV-NDMT group maintained its weight. These results suggest that BMV-NDMT can maintain the mice healthier. Tumors consume large amounts of glucose and amino acids, which causes protein degradation in skeletal muscle and reduction of adipose tissue mass due to lipase activation, generating an energy imbalance.^47^

Tumor growth in mice treated with BMV-NDMT is shown in Figure 6A. Although all groups showed an increase in tumor volume over time, mice treated with BMV-NDMT had significantly smaller tumors starting on day 17, at the time of the third dose of treatment. At the end of the experiment, tumor volume in the BMV-NDMT group was 44% smaller (*p* = 0.001) than in the PBS group. Tumors treated with BMV showed a 19% smaller volume (*p* = 0.0304) than those in the PBS group. ANOVA analysis indicated significant differences between groups (*p* = 0.003), suggesting that BMV-NDMT has more than two-times more antitumor effect than BMV alone. Previous studies have shown that BMV can induce antitumor responses, although the exact mechanism remains unclear. Virus-like particles (VLPs) can trigger immune responses, such as leukocyte recruitment and T and B lymphocyte activation, contributing to tumor and metastatic inhibition.^34,48^

**Figure 6:**
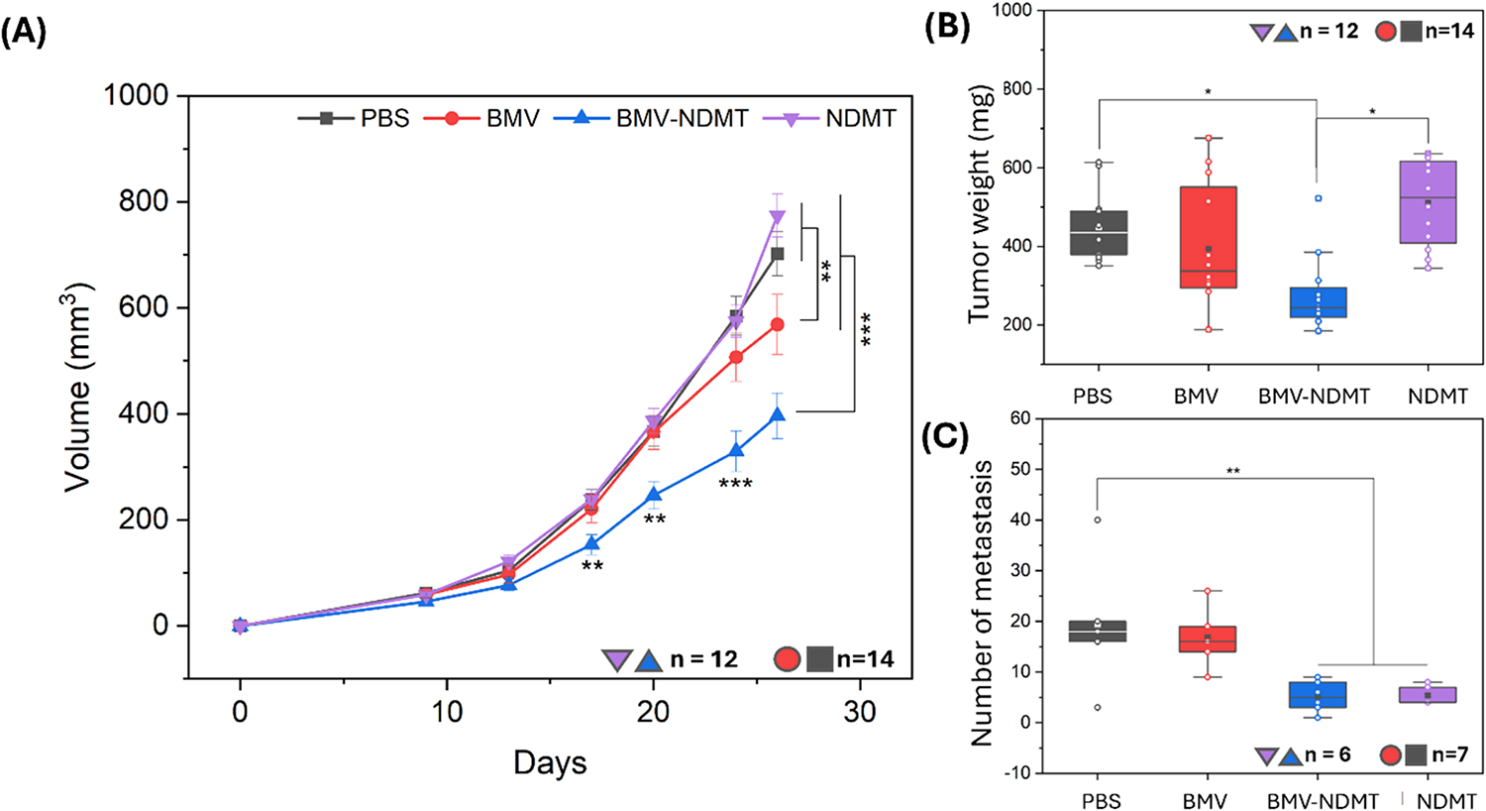
4T1 Tumors and lung metastases: (A) Volume of 4T1 tumors in Balb/c mice. (B) Weight of 4T1 tumors removed from Balb/c mice on day 27 (for A and B, *n* = 12 for NDMT and BMV, *n* = 14 for PBS and BMV-NDMT). (C) Number of metastases found on the lungs of Balb/c mice (*n* = 6 for NDMT and BMV, *n* = 7 for PBS and BMV-NDMT). In the graphs, the results from BMV-NDMT are marked in blue, and control experiments BMV in red, NDMT in purple and PBS in black. Points represent the average viability, and error bars represent the standard error of mean (SEM). Statistical analyses One-way ANOVA, Tukey test, *P < 0.01, **P < 0.05, ***P < 0.005.

Mice were sacrificed on day 27 to remove tumors and lungs. Figure 6B shows the tumor weights of the groups treated with BMV-NDMT (blue), PBS (black), BMV (red), and NDMT (purple). Tumors in the control groups (PBS, BMV and NDMT) ranged from 200 to 700 mg, with no significant differences between them. However, tumors in the BMV-NDMT group weighed between 200 and 400 mg, being significantly smaller than those in the PBS (*p* = 0.0024) and NDMT groups (*p* = 0.000059). The images of the extracted tumors are shown in Figure S8B; the tumors from mice treated with BMV-NDMT were smaller, consistent with the known antiproliferative and proapoptotic effects of tamoxifen and its metabolites, such as NDMT in MDA-MB-231 cells, mediated by the activation of the JNK1 (c-Jun N-terminal kinase-1) pathway, which induces apoptosis through interference with glycosphingolipid metabolism.^49^

Although multiple nanocarriers have been developed for the controlled administration of tamoxifen, reports on delivery systems for its metabolite NDMT are scarce. Sreekanth et al. synthesized cholic acid-NDMT conjugates and observed 50% tumor growth inhibition in BALB/c mice bearing 4T1 tumors using an NDMT dose of 15 mg/kg (approximately 300 μg).^50^ In the present study, BMV-NDMT achieved a comparable tumor inhibition of 44% at a significantly lower dose of 0.125 mg/kg (2.5 μg of NDMT). This represents a 120-fold reduction in the required drug amount to achieve a similar antitumor effect. Furthermore, BMV-NDMT VNPs showed similar activity in MDA-MB-231 cells (IC_50_ 4.95 ± 0.42 ng/μL) to the cholic acid–based nanovehicles (IC_50_ 6.25 ng/μL).^50^ Crucially, this comparable *in vivo* efficacy was achieved with a 6-fold lower NDMT dose (2.5 mg/kg *vs* 15 mg/kg), suggesting greater therapeutic efficacy of the VNP platform.

### Lung Metastasis

Lung tissues are one of the most common sites where tumor cells spread in advanced stages of cancer.^51^ Lung metastasis in BALB/c mice inoculated with 4T1 cells was determined by perfusing the lungs with Indian ink to visualize tumor nodules. Figure 6C shows the number of tumor nodules due to the metastasis in the lungs of mice treated with PBS (19.4 ± 13.29), BMV (16.8 ± 6.30), NDMT (5.4 ± 1.94), and BMV-NDMT (5.16 ± 3.06). ANOVA analysis revealed significant differences between the PBS group and the NDMT (*p* = 0.036) and BMV-NDMT (*p* = 0.025) groups. Figure S8C,D show images of lung lobes without metastasis and with metastasis, respectively. These results suggest that both NDMT and BMV-NDMT significantly reduce the onset of metastasis to the lungs in mice with 4T1 tumors. Metastasis involves the loss of adhesion proteins in tumor cells, which increases their invasiveness and mobility, allowing them to detach from the primary tumor, enter the bloodstream, and form secondary tumors in distant tissues.^52^ Metastasis is responsible for the majority of deaths from breast cancer, the second most common cancer worldwide after lung cancer and the most common among women, accounting for 23.8% of all female cancer cases. In 2020, approximately 685,000 women died from this disease, and its global incidence continues to rise. Given that delaying or preventing metastasis could improve patient survival by up to 84%,^1^ strategies to inhibit this process are critical. In this study, BMV-NDMT VNPs reduced the development of lung metastasis by 74.4%, highlighting their potential to inhibit metastatic spread.

## Conclusion

This study successfully establishes plant virus-derived nanoparticles, particularly from BMV, as a highly efficient platform for the delivery of N-desmethyl-tamoxifen (NDMT) to treat triple-negative breast cancer. Our work demonstrates that the structural and physicochemical properties of BMV confer a superior NDMT loading capacity compared to CCMV, a finding predicted by *in silico* simulations and confirmed experimentally. The resulting BMV-NDMT complexes were efficiently internalized by cancer cells, localizing to both the cytoplasm and the nucleus, and exhibited significantly enhanced cytotoxicity *in vitro* compared to the free drug.

The high level of importance of this work is underscored by our *in vivo* results, where BMV-NDMT therapy achieved a 44% reduction in tumor volume and a 74% reduction in lung metastasis at an exceptionally low dose of 2.5 μg of NDMT per administration. This represents a 6-fold lower dose than that required in previous studies using other NDMT delivery systems to achieve a comparable antitumor effect.^40^ This dose reduction is a critical advance, as it directly translates to a potentially vastly improved safety profile and reduced risk of off-target side effects, addressing a major hurdle in cancer chemotherapy.

This work lays the foundation for several promising future directions. First, the mechanisms behind the impressive antimetastatic activity warrant deeper investigation. Second, future studies should explore the targeted delivery of VNPs by conjugating targeting ligands (*e.g.*, peptides, antibodies) to the viral capsid to further enhance specificity for tumor tissue. Finally, the platform’s versatility should be exploited to encapsulate other hydrophobic drugs or combination therapies, expanding its utility beyond NDMT.

## Methods

### Production, Purification, and Characterization of BMV and CCMV

Brome mosaic virus (BMV) and cowpea chlorotic mottle virus (CCMV) were propagated on barley (*Hordeum vulgare*) and cowpea (*Vigna unguiculata*) plants, respectively. After 2 weeks of planting germinated seeds, their first leaves were infected with a viral solution (0.1 mg/mL) in inoculation buffer (0.01 M sodium phosphate, 0.01 M magnesium chloride, pH 6.0). Three weeks later, the leaves showing chlorosis symptoms were harvested and stored at −20 °C for further processing. For virus purification, 250 g of infected leaves were ground in the extraction solution (0.5 M sodium acetate, 0.08 M magnesium acetate, pH 4.5, 2% v/w β-mercaptoethanol) using a blender. The homogenate was filtered through a cheesecloth to remove plant debris. Subsequently, 200 mL of cold chloroform was added to the sample and stirred for 10 min at 4 °C. Then, the solution was centrifuged at 10,000 rpm for 20 min at 4 °C in a JA-14 rotor using a Beckman centrifuge (JXN-26). To precipitate the viral particles, 10% (w/v) PEG (8000MW) was added to the supernatant and stirred for 12 h at 4 °C. The solution was centrifuged at 10,000 rpm for 20 min, and the obtained pellet was resuspended in SAMA buffer (0.05 M sodium acetate, 0.008 M magnesium acetate, pH 4.5). To purify the viruses, the sample was ultracentrifuged over a 10% sucrose cushion at 32,000 rpm for 2 h and 30 min at 4 °C, using SW-32 Ti rotor in an XPN100 ultracentrifuge (Beckman). The resulting pellet was resuspended in SAMA buffer stored at −80 °C until use. The concentration and purity of the viruses were determined by UV–vis spectrophotometry (Nanodrop 200c, Thermo Scientific), using eqs 2 and 3, respectively. In these equations, A represents absorbance, CE is the extinction coefficient, 5.15 for BMV and 5.8 for CCMV. The hydrodynamic diameter of the viral particles was measured by dynamic light scattering (DLS) using a Zetasizer NanoZS (Malvern Instruments Ltd.). Virions were stained with 2% uranyl acetate on carbon-coated copper grids to observe their morphology by transmission electron microscopy (TEM, HF-3300, Hitachi). TEM was operated at 100 keV, and digital images were captured in brightfield mode at magnifications of 40,000× and 70,000×. The TEM micrographs were analyzed using ImageJ 2 software. Particle size distributions were obtained by measuring a minimum of 100 viral nanoparticles from the digital TEM images.

2 $$C_{v}=\frac{A260}{\mathrm{EC}}$$

3 $$P_{v}=\frac{A260}{A280}∼1.8$$

### Theoretical Quantification of *N*-Desmethyl-Tamoxifen

The theoretical number of NDMT molecules that can dock to the capsid proteins of BMV and CCMV was determined by molecular docking simulations using Autodock Vina 1.2 software (https://vina.scripps.edu/). Molecular interactions between NDMT and viral proteins were analyzed and visualized using PyMOL 3.1.3 (https://pymol.org/), and LigPlot+ 2.2.9 (https://www.ebi.ac.uk/thornton-srv/software/LigPlus/).The three-dimensional (3D) model of the NDMT molecule was obtained from the PubChem database (ID: 6378383).^53^ The structures of the BMV (ID: 3j7l) and CCMV (ID: 1za7) capsid proteins were obtained from the Protein Data Bank (PDB).^54,55^ These structures were prepared for molecular docking by removing ligands and water molecules and adding atomic charges. The amount of NDMT binds to the capsid protein was calculated using the eq 4.

4 $$g\;{\left(m\right)}_{f}=\frac{g_{v}\times P_{v}\times M_{pp}\times 180}{{PM}_{f}}$$

Where *g*_v_ is the mass of the virus (grams), *P*_v_ is the molecular weight of the viruses (approximate 4.6 × 10^6^ g/mol), *M*_pp_ is the NDMT molecules docked per protein, and PM_f_ is the molecular weight of NDMT (357.5 g/mol).

### Synthesis and Purification of *N*-Desmethyl-Tamoxifen

Synthesis of NDMT was carried out from tamoxifen by modifications of previous reported protocol.^13^ The synthesis scheme is illustrated in Figure 7. In a three-neck round-bottom flask, protected from light, 430 mg of tamoxifen (Sigma-Aldrich, CAS 68047–06–3) were dissolved in 8 mL of 1,2-dichloroethane and kept under constant stirring in an ice bath at 0 °C. Subsequently, 1-chloroethyl chloroformate was added in a molar ratio of 1:1 with respect to tamoxifen, keeping the mixture at 0 °C and stirring. After 15 min, the reaction was refluxed for 24 h under a constant nitrogen flow. Afterward, the solvent was evaporated using a rotary evaporator. Then, 15 mL of methanol was added to the obtained oily product, and the solution was refluxed again for 24 h, at 70 °C, under inert conditions and protection from light. Finally, the solvent was evaporated to obtain NDMT as an off-white powder. It was dissolved in 2 mL of 3% dichloromethane/methanol and passed through a silica gel grade 633 column using the same solution as eluent. Aliquots of 5 mL were collected and analyzed by thin-layer chromatography (TLC), the fractions corresponding to NDMT were collected and dehydrated with sodium sulfate, filtered (0.45 μm), until obtaining a white powder (∼64% yield). The successful synthesis of NDMT was characterized by FTIR and high-resolution mass analysis (HR-ESI–MS: calculated mass for C_25_H_27_NO, 357.50 m, and found [M + H]^+^ 358.2195 *m*/*z*). In addition, NDMT was characterized by fluorescence spectroscopy to determine its excitation and emission spectra using a fluorimeter (Cary Eclipse G9800A, Agilent). Also, a fluorescence calibration curve as a function of NDMT mass was obtained.

**Figure 7:**
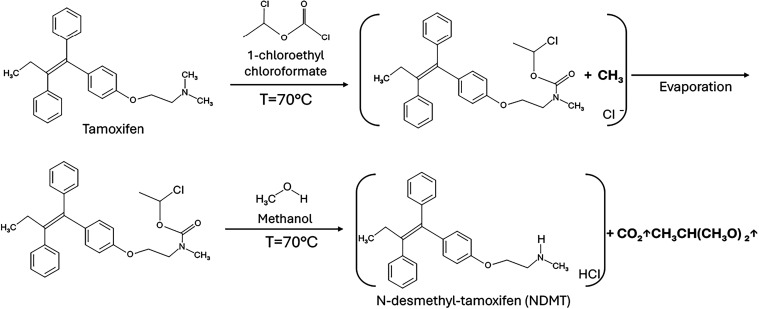
*N*-Desmethyl-tamoxifen synthesis reaction: Acylation reaction of the dimethylamine group of tamoxifen (top), deacylation reaction of the dimethylamine group by hydrogen substitution.

### *N*-Desmethyl-Tamoxifen Loading into BMV and CCMV

BMV and CCMV were changed to a phosphate-buffered saline (PBS) solution by ultrafiltration using Ultracel 30 kDa MWCO filters (Millipore). For drug loading, 200 μg of virus (40 μL) was mixed with 40 μg of NDMT dissolved in 40 μL of DMSO, maintaining a 1:1 ratio of PBS and DMSO. The mixture was incubated in darkness with gentle shaking for 1 h at 4 °C. VNPs loaded with NDMT were then purified by ultrafiltration to remove the DMSO and excess unbound drug. VNPs in PBS were recovered from the filter and stored, protected from light, at 4 °C. The VNPs were characterized by DLS and TEM and quantified by fluorescence spectroscopy. The amount of NDMT coupled to the virus was calculated using equations derived from calibration curves obtained by fluorescence spectroscopy.

The amount of NDMT coupled to the virus was quantified by fluorescence spectroscopy using a standard curve of free NDMT. While it is acknowledged that the fluorescent properties of NDMT (*e.g.*, quantum yield) could be altered upon binding to the capsid protein or within the viral interior, the strong correlation between these experimental results and the *in silico* docking predictions supports the validity of this quantitative approach. The standard curve was constructed in a DMSO/PBS mixture to counteract the hydrophobicity of the drug and approximate the hydrophobic environment within the capsid.

### Cell Culture

The MDA-MB-231 and 4T1 cell lines, representative models of triple-negative breast cancer (HR^–^/ERBB2^–^), were used to evaluate the efficacy of viral nanoparticles (VNPs) loaded with NDMT. MDA-MB-231 cells, derived from a metastatic human breast adenocarcinoma, and 4T1 cells, originating from a mouse mammary tumor of the BALB/cfC3H strain, were cultured in 6 cm dishes. MDA-MB-231 cells were maintained in DMEM medium (Sigma-Aldrich), while 4T1 cells were cultured in the RPMI medium (Sigma-Aldrich). Both media were supplemented with 10% (v/v) fetal bovine serum (FBS, Biowest) and 1% (v/v) antibiotic/antimycotic (Corning Cellgro). Cultures were incubated at 37 °C in a 5% CO_2_ atmosphere. When cells reached 80% confluence, subcultures were performed using 0.05% trypsin (Corning Cellgro). For preservation, cells were frozen at −80 °C in a complete medium with 10% (v/v) DMSO at a concentration of 1 million cells per mL.

### Cell internalization of VNPs

To evaluate the internalization of viral nanoparticles (VNPs) loaded with NDMT, they were labeled with NanoOrange (Invitrogen), a hydrophobic fluorophore that specifically binds to the hydrophobic domains of capsid proteins. NanoOrange has an excitation and emission maxima at 470 and 570 nm, respectively. Labeling and purification of VNPs were performed following previous protocol.^34^ Briefly, VNPs were incubated with NanoOrange for 20 min to allow fluorophore binding. Excess fluorophore was removed by ultrafiltration using 100 kDa Amicon filters (0.5 mL, Millipore).

Then, 100,000 cells (synchronized in the G0/G1 phase of the cell cycle) were seeded on glass coverslips previously coated with poly-2-lysine to improve cell adhesion. Subsequently, NanoOrange-labeled VNPs were added and incubated for 4 h at 37 °C in a 5% CO_2_ atmosphere. After incubation, cells were fixed with 4% glutaraldehyde in PBS and stored at −20 °C, protected from light, for 20 min. The cytoskeleton was labeled with Phalloidin CruzFluor 594 Conjugate (exc. 590 nm), 1:100 in PBS-Tween (0.3% v/v), and cell nuclei were stained with DAPI (0.1 μg/mL, exc. 405 nm). Coverslips were mounted on slides using PBS with glycerol 1:1 (v/v). Samples were visualized using an FV1000 FluoView confocal microscope (Olympus) with a 60x objective and a numerical aperture of 1.42. Images were acquired at excitation wavelengths corresponding to each fluorophore, allowing visualization of the cytoskeleton, cell nuclei, and internalized VNPs. To process the images and analyze them we use Fiji an image processing package from ImageJ.

### Cell Viability Assay

The cytotoxicity of viral nanoparticles (VNPs) loaded with NDMT was assessed by using the *in vitro* toxicology assay KIT MTT based (Sigma-Aldrich), which measures cellular metabolic function through the reduction of 3-(4,5-dimethylthiazol-2-yl)-2,5-diphenyltetrazolium bromide (MTT) to formazan. For this assay, 20,000 cells in complete medium were seeded per well in 96-well plates. Cells were treated with different concentrations of VNPs and incubated for 24 h at 37 °C under a 5% CO_2_ atmosphere. Subsequently, 10 μL of MTT solution (5 mg/mL) was added to each well and incubated for 4 h at 37 °C under 5% CO_2_. The formed formazan crystals were solubilized by adding 100 μL of lysis solution. Finally, the absorbance at 570 nm was measured using an ELISA plate reader. Cell viability was calculated by comparing the absorbance for treated cells compared with untreated control cells.

Doses are reported in terms of NDMT concentration. Based on the loading capacity determined in Table 1, these concentrations can be converted to viral nanoparticle (VNP) concentration. For reference, the IC_50_ of BMV-NDMT in MDA-MB-231 cells (4.95 ng/μL NDMT) corresponds to a VNP concentration of approximately 36.75 ng/μL. The virus and drug concentrations used to formulate the VNPs at the IC50 concentration are detailed in Table S1.

### Animal Experiment

Animal experiments were conducted in accordance with the Mexican Official Standard NOM-062-ZOO-1999 (SAGARPA, Mexico City, Mexico) and were approved by the Institutional Bioethics Committee of the Center of Scientific Research and Higher Education of Ensenada (CICESE) (protocol code ANIM_TERR_2020_01, approved 02/25/2020). Four-week-old, female BALB/cAnNHsd mice were purchased from Circulo-ADN. Animals were housed in an Optimice cage system (Animal Care Systems) in a controlled environment (24 °C, 12-h light/dark cycle) with free access to water and food (Laboratory Autoclavable Rodent Diet, 24% protein content; LabDiet). Mice were acclimatized for at least 1 week before starting the experiments.

To induce tumors, a suspension of 4T1 breast cancer cells was prepared at a concentration of 2 × 10^6^ cells/mL in PBS. Then, 10^5^ cells in 50 μL of PBS were inoculated into both lower mammary fat pads (To obtain two tumors per mice) of 5–6 week-old female BALB/c mice using a 300 μL insulin syringe with a 29 G needle. One week after the inoculation, the formation of palpable tumor was confirmed in all mice. Animals were divided into four groups (*n* = 6 for NDMT and BMV, *n* = 7 for PBS and BMV-NDMT) to receive the following treatments: BMV-NDMT, NDMT, BMV, and PBS. Treatments were administered intratumorally twice weekly by injecting 25 μL of VNPs at a concentration of 4 μg/μL (equivalent to 2.5 μg of drug), NDMT (2.5 μg), BMV (100 μg), or PBS. Treatments were applied six times in total.

Tumor size was measured three times per week using a caliper and was calculated using the formula (*L* × *w* ^2^)/2, where *L* and *w* represent tumor length and width, respectively. Mice were sacrificed on day 27 postinoculation. Tumors were removed and weighed. To assess the spontaneous formation of lung metastasis from the mammary fat pad tumors, mouse lungs were perfused with Indian ink (15% v/v) and fixed in a neutral buffered paraformaldehyde solution. Lungs were then dissected into lobes and examined under a stereoscope to count white protuberances, indicative of metastasis.

### Statistical Analysis

The comparison of the treatments in cell viabillity, 4T1 Tumors and lung metastases was evaluated by Shapiro-Wilk using the “Shapiro-Wilk Test Calculator” available on “Statistics Kingdom” (https://www.statskingdom.com/shapiro-wilk-test-calculator.html). One-way ANOVA and Tukey’s test was evaluated using VassarStats of Vassar College, NY-USA (http://faculty.vassar.edu/lowry/anova1u.html).

## Supplementary Material



## Notes

Types of interactions between the BMV protein and NDM-tamoxifen; structural characterization of the NDM-tamoxifen molecule; emission spectra of NDM-tamoxifen; virus characterization; alignment of the amino acid sequences of CCMV and BMV; emission of NanoOrange-labeled VNPs; dose–response curves of VNP concentrations and IC_50_ values; and photographs of tumors and lung metastases in mice (PDF)

## References

[1] Filho A. M., Laversanne M., Ferlay J., Colombet M., Piñeros M., Znaor A., Parkin D. M., Soerjomataram I., Ervik M., Bray F. (2025). The GLOBOCAN 2022 cancer estimates:
Data sources, methods, and a snapshot of the cancer burden worldwide. Int. J. Cancer.

[2] Waks A. G., Winer E. P. (2019). Breast cancer treatment: a review. JAMA.

[3] Lumachi F., Brunello A., Maruzzo M., Basso U., Mm Basso S. (2013). Treatment
of estrogen receptor-positive breast cancer. Curr. Med. Chem..

[4] Mirfakhraee S., Chan A. V. C., Ganji N., Abramowitz J. (2021). Dual treatment
of acromegaly and hormone-receptor-positive breast cancer with tamoxifen:
a case report. J. Med. Case Rep..

[5] Shagufta, Ahmad I. (2018). Tamoxifen a pioneering drug: An update
on the therapeutic potential of tamoxifen derivatives. Eur. J. Med. Chem..

[6] Trujillo-Martínez M., Gómez-Flores-Ramos L., Sánchez-Zamorano L. M., Reynoso-Noverón N., Grimaldo L., Albavera-Hernández C., Flores-Luna L. (2022). Farmacogenética
en el cáncer de mama: implicaciones de los genes del citocromo
p450 en la supervivencia libre de la enfermedad en las mujeres jóvenes. Rev. Senol. Patol. Mamar..

[7] Lu W. J., Desta Z., Flockhart D. A. (2012). Tamoxifen
metabolites as active inhibitors of aromatase in the treatment of
breast cancer. Breast Cancer Res. Treat..

[8] Lien E. A., Solheim E., Ueland P. M. (1991). Distribution of tamoxifen and its metabolites in rat
and human tissues during steady-state treatment. Cancer Res..

[9] Bobin-Dubigeon C., Campone M., Rossignol E., Salaun E., Amiand M. B., Bard J. M. (2019). New UPLC–MS/MS assay for the determination of
tamoxifen and its metabolites in human plasma, application to patients. Future Sci. OA.

[10] Wu X., Xiong H. (2024). The Role of Pharmacogenetic-Based
Pharmacokinetic Analysis in Precise Breast Cancer Treatment. Pharmaceutics.

[11] Chung Y. H., Cai H., Steinmetz N. F. (2020). Viral nanoparticles
for drug delivery, imaging, immunotherapy, and theranostic applications. Adv. Drug Delivery Rev..

[12] Kumar M., Sharma G., Misra C., Kumar R., Singh B., Katare O. P., Raza K. N. (2018). desmethyl tamoxifen
and quercetin-loaded multiwalled CNTs: A synergistic approach to overcome
MDR in cancer cells. Mater. Sci. Eng.: C.

[13] Dreaden E. C., Mwakwari S. C., Sodji Q. H., Oyelere A. K., El-Sayed M. A. (2009). Tamoxifen– poly (ethylene
glycol)– thiol gold nanoparticle conjugates: enhanced potency
and selective delivery for breast cancer treatment. Bioconjugate Chem..

[14] González-Davis O., Villagrana-Escareño M. V., Trujillo M. A., Gama P., Chauhan K., Vazquez-Duhalt R. (2023). Virus-like
nanoparticles as enzyme carriers for enzyme replacement therapy (ERT). Virology.

[15] Deo V. K., Kato T., Park E. Y. (2015). chimeric virus-like
particles made using GAG and M1 capsid proteins providing dual drug
delivery and vaccination platform. Mol. Pharmaceutics.

[16] Loredo-García E., Herrera-Hernandez M. M., Medrano-Villagómez C., Fournier P. G. J., Rodriguez-Hernandez A. G., Loredo-Tovías M., Ruiz-Garcia J., Dragnea B., Cadena-Nava R. (2026). The solvent
stability of bromovirus allows for delivery of hydrophobic chemotherapeutic
drugs. Mater. Adv..

[17] Duran Meza A. L., Porak S., Chapman A., Knobler C. M., Gelbart W. M. (2025). How much genetic information in RNA
form can be protected by a CCMV virus-like particle?. PLoS One.

[18] Villagrana-Escareño M. V., Reynaga-Hernández E., Galicia-Cruz O. G., Durán-Meza A.
L., De la Cruz-González V., Hernández-Carballo C., Ruíz-García J. (2019). VLPs Derived
from the CCMV Plant Virus Can Directly Transfect and Deliver Heterologous
Genes for Translation into Mammalian Cells. BioMed Res. Int..

[19] Malyutin A. G., Cheng H., Sanchez-Felix O., Carlson K., Stein B. D., Konarev P. V., Svergun D. I., Bogdan D., Bronstein L. M. (2015). Coat Protein-Dependent
Behavior of Poly­(ethylene glycol) Tails in Iron Oxide Core Virus-like
Nanoparticles. ACS Appl. Mater. Interfaces.

[20] Bruckman M. A., Czapar A. E., VanMeter A., Randolph L. N., Steinmetz N. F. (2016). Tobacco mosaic virus-based protein
nanoparticles and nanorods for chemotherapy delivery targeting breast
cancer. J. Controlled Release.

[21] Le D. H. T., Lee K. L., Shukla S., Commandeur U., Steinmetz N. F. (2017). Potato virus X, a filamentous plant
viral nanoparticle for doxorubicin delivery in cancer therapy. Nanoscale.

[22] Pitek A. S., Hu H., Shukla S., Steinmetz N. F. (2018). Cancer theranostic applications of albumin-coated tobacco
mosaic virus nanoparticles. ACS Appl. Mater.
Interfaces.

[23] Bijari N., Ghobadi S., Derakhshandeh K. (2019). β-lactoglobulin-irinotecan
inclusion complex as a new targeted nanocarrier for colorectal cancer
cells. Res. Pharm. Sci..

[24] Duran-Meza A. L., Villagrana-Escareño M. V., Ruiz-García J., Knobler C. M., Gelbart W. M. (2021). Controlling the
surface charge of simple viruses. PLoS One.

[25] Dill K. A., Bromberg S., Yue K., Chan H. S., Ftebig K. M., Yee D. P., Thomas P. D. (1995). Principles of protein
folding—a perspective from simple exact models. Protein Sci..

[26] Xie L., Yang D., Lu Q., Zhang H., Zeng H. (2020). Role of molecular
architecture in the modulation of hydrophobic interactions. Curr. Opin. Colloid Interface Sci..

[27] Almeida F. C. L., Sanches K., Pinheiro-Aguiar R., Almeida V. S., Caruso I. P. (2021). Protein surface interactions—theoretical
and experimental studies. Front. Mol. Biosci..

[28] Van Oss C. J., Good R. J., Chaudhury M. K. (1986). The role
of van der Waals forces and hydrogen bonds in “hydrophobic
interactions” between biopolymers and low energy surfaces. J. Colloid Interface Sci..

[29] Antunes M. V., Raymundo S., de Oliveira V., Staudt D. E., Gössling G., Peteffi G. P., Villanova J., Cavalheiro J. A., Tre-Hardy M., Capron A., Haufroid V., Wallemacq P., Schwartsmann G., Linden R. (2015). Ultra-high performance
liquid chromatography tandem mass spectrometric method for the determination
of tamoxifen, N-desmethyltamoxifen, 4-hydroxytamoxifen and endoxifen
in dried blood spots—development, validation and clinical application
during breast cancer adjuvant therapy. Talanta.

[30] Adamczyk J., Brzozowska-Rup K., Sieroń D., Sieroń K., Sieroń A. (2024). Fluorescence
spectral analysis and logistic regression modeling for diagnosing
basal cell carcinoma on head and neck. Photodiagn.
Photodyn. Ther..

[31] Zeng H., Korbelik M., McLean D. I., MacAulay C., Lui H. (2002). Monitoring Photoproduct Formation and Photobleaching by Fluorescence
Spectroscopy Has the Potential to Improve PDT Dosimetry with a Verteporfin-like
Photosensitizer. Photochem. Photobiol..

[32] Xie A., Tsvetkova I., Liu Y., Ye X., Hewavitharanage P., Dragnea B., Cadena-Nava R. (2021). Hydrophobic Cargo Encapsulation into
Virus Protein Cages by Self-Assembly in an Aprotic Organic Solvent. Bioconjugate Chem..

[33] Bujarski J., Gallitelli D., García-Arenal F., Pallás V., Palukaitis P., Reddy M. K., Wang A., ICTV Report Consortium (2019). ICTV virus taxonomy profile: Bromoviridae. J. Gen. Virol..

[34] Nuñez-Rivera A., Fournier P. G., Arellano D. L., Rodriguez-Hernandez A. G., Vazquez-Duhalt R., Cadena-Nava R. D. (2020). Brome mosaic virus-like particles
as siRNA nanocarriers for biomedical purposes. Beilstein J. Nanotechnol..

[35] Tejeda-Rodríguez J. A., Nunez A., Soto F., García-Gradilla V., Cadena-Nava R., Wang J., Vazquez-Duhalt R. (2019). Virus-Based Nanomotors for Cargo
Delivery. ChemNanoMat.

[36] Tao K., Fang M., Alroy J., Sahagian G. G. (2008). Imagable 4T1 model for the study of late stage breast
cancer. BMC Cancer.

[37] Wu W., Hsiao S., Carrico Z. (2009). Genome-free viral capsids as multivalent carriers for taxol delivery. Angew. Chem., Int. Ed..

[38] Su H., Yang F., Sun B., Karin M. (2021). Macropinocytosis: the
big drinker behind cancer cell self-consumption. Autophagy.

[39] Butler L. M., Perone Y., Dehairs J., Lupien L. E., de Laat V., Talebi A., Swinnen J. V. (2020). Lipids
and cancer: Emerging roles in pathogenesis, diagnosis and therapeutic
intervention. Adv. Drug Delivery Rev..

[40] Du Y., Kong G., You X., Zhang S., Zhang T., Gao Y., Ye L., Zhang X. (2012). Elevation of highly up-regulated in liver cancer (HULC) by hepatitis
B virus X protein promotes hepatoma cell proliferation via down-regulating
p18. J. Biol. Chem..

[41] Omole A. O., Newton H. S., Cedrone E., Nematpour K., Xie S., Zhao Y., Steinmetz N. F. (2025). Comparative analyses
for plant virus-based cancer immunotherapy drug development. Cell Biomater..

[42] Su H., Yang F., Sun B., Karin M. (2021). Macropinocytosis: the big drinker behind cancer cell self-consumption. Autophagy.

[43] Wu W., Hsiao S. C., Carrico Z. M., Francis M. B. (2009). Genome-free viral capsids as multivalent carriers for
taxol delivery. Angew. Chem., Int. Ed..

[44] Lu W. J., Desta Z., Flockhart D. A. (2012). Tamoxifen metabolites as active inhibitors of aromatase
in the treatment of breast cancer. Breast Cancer
Res. Treat..

[45] Licznerska B. E., Szaefer H., Murias M., Bartoszek A., Baer-Dubowska W. (2013). Modulation of CYP19 expression by cabbage juices and
their active components: indole-3-carbinol and 3, 3′-diindolylmethene
in human breast epithelial cell lines. Eur.
J. Nutr..

[46] Manna S., Holz M. K. (2016). Tamoxifen action in ER-negative breast
cancer. Signal Transduction Insights.

[47] Argilés J. M., Busquets S., Stemmler B., López-Soriano F. J. (2014). Cancer cachexia: understanding the
molecular basis. Nat. Rev. Cancer.

[48] Lizotte P.
H., Wen A. M., Sheen M. R., Fields J., Rojanasopondist P., Steinmetz N. F., Fiering S. (2016). In situ vaccination with cowpea mosaic
virus nanoparticles suppresses metastatic cancer. Nat. Nanotechnol..

[49] Gundimeda U., Chen Z. H., Gopalakrishna R. (1996). Tamoxifen modulates protein kinase
C via oxidative stress in estrogen receptor-negative breast cancer
cells. J. Biol. Chem..

[50] Sreekanth V., Bansal S., Motiani R. K., Kundu S., Muppu S. K., Majumdar T. D., Panjamurthy K., Sengupta S., Bajaj A. (2013). Design, synthesis, and mechanistic
investigations of bile acid–tamoxifen conjugates for breast
cancer therapy. Bioconjugate Chem..

[51] Hu H., Wang J., Wang H., Tan T., Li J., Wang Z., Sun K., Li Y., Zhang Z. (2018). Cell-penetrating peptide-based nanovehicles potentiate lymph metastasis
targeting and deep penetration for anti-metastasis therapy. Theranostics.

[52] Cavallaro U., Christofori G. (2001). Cell adhesion
in tumor invasion and metastasis: loss of the glue is not enough. Biochim. Biophys. Acta, Rev. Cancer.

[53] Desmethyltamoxifen (PubChem CID: 6378383, CAS RN: 31750–48–8). PubChem. U.S. Department of Health and Human Services, National Library of Medicine, National Center for Biotechnology Information. https://pubchem.ncbi.nlm.nih.gov/compound/6378383.

[54] Wang Z., Hryc C. F., Bammes B., Afonine P. V., Jakana J., Chen D. H., Liu X., Baker M. L., Kao C., Ludtke S. J., Schmid M. F., Adams P. D., Chiu W. (2014). An atomic model of brome mosaic virus
using direct electron detection and real-space optimization. Nat. Commun..

[55] Speir J. A., Bothner B., Qu C., Willits D. A., Young M. J., Johnson J. E. (2006). Enhanced local symmetry interactions globally stabilize
a mutant virus capsid that maintains infectivity and capsid dynamics. J. Virol..

