# Supplementary material for: BMV and CCMV-Based Viral Nanoparticles for Delivery of N‑Desmethyl-Tamoxifen as Treatment of Triple-Negative Breast Cancer
Source: ACS Omega. 2026 Feb 27;11(9):14976–88. doi: 10.1021/acsomega.5c11566 (PMC12980261; doi:10.1021/acsomega.5c11566)
Supplement: Supplementary file 1 [file ao5c11566_si_001.pdf]

BMV and CCMV-based viral nanoparticles for delivery of N-desmethyl-tamoxifen as treatment of triple-negative breast cancer

Elizabeth Loredo-García [a,b], Pierrick G. J. Fournier [b], M. Mariana Herrera-Hernandez [a,b], Kanchan Chauhan [a], Ana G. Rodriguez-Hernandez [a], Rafael Vazquez-Duhalt [a], Ruben D. Cadena-Nava [a,*].

[a] Centro de Nanociencias y Nanotecnología, Universidad Nacional Autónoma de México (UNAM), Km. 107 Carretera Tijuana-Ensenada, Ensenada, Baja California, CP. 22800, México

[b] Centro de Investigación Científica y de Educación Superior de Ensenada, Baja California, (CICESE), Carretera Ensenada-Tijuana 3918, Ensenada, Baja California, CP. 22860, México.

* Email: rcadena@ens.cnyn.unam.mx

Supplementary Material

Theoretical quantification of NDM-tamoxifen

Table S1. Interactions between BMV protein and NDM-tamoxifen[a]

Pocket BMV-NDMT	Affinity energy (kCal/mol)	Hydrophobic interactions (#70) 85.3%		Hydrogen bonds (#12) 14.63%	
P1_BMV_NDMT	-5.6	6	C9/Leu94, C15/Ala134, C15/Trp93, C18/Val133, C23/Ala37, C24/Gly38	3	N/Gln39, N/Gly40, N/Ala36
P2_BMV_NDMT	-5.5	9	N/Trp54, O/Pro74, C12/Ile51, C19/Glu76, C20/Phe180, C20/Prol178, C22/Ser52, C22/Leu77, C25/Glu55	1	N/Lys53
P3_BMV_NDMT	-5.5	9	O/Ile51, C2/Glu172, N/Arg89, C15/Ile43, C15/Lys44, 16/Ala42, C20/Trp93, C25/Val90, C25/Leu91	1	N/Glu174
P4_BMV_NDMT	-5.4	3	C16/Pro186, C20/Phe183, C25/Asp182	1	N/Phe184
P5_BMV_NDMT	-5.4	5	C6/Gln120, C14/Lys105, C14/Tyr157, C22/Glu116, C25/Val125	1	N/Phe119
P6_BMV_NDMT	-5.2	9	C9/Leu123, C9/Val121, C10/Ala122, C19/Tyr137, C21/Phe141, C21/Val108, C22/Glu110, C25/Ala144, C25/Arg142	1	N/Ala140
P7_BMV_NDMT	-5.0	7	N/Tyr137, O/Ala124, C18/Ala134, C19/Asp127,	2	N/Thr138, N/Met136

			C19/Val125, C19/Val132, C20/Lys130		
P8_BMV_NDMT	-5.0	5	C5/Phe180, C6/Glu76, C16/His75, C21/Ser79, C25/Pro178	1	N/Asp181
P9_BMV_NDMT	-4.7	6	C19/Lys83, C20/Ser78, C21/Leu73, C23/Leu150, C25/Asp151, C25/Lys111	0	-
P10_BMV_NDMT	-5.3	11	C9/Ala65, C5/Thr66, C15/Glu114, C13/Glu116, C21/Asn67, C21/Thr62, C24/Ala113, C24/Ala115, C24/Glu112, C25/Ala68, C25/Lys111	1	N/Tyr155

[a]Affinity energies and interactions of drug-capsid protein obtained by molecular docking with Autodock Vina. Amino acids are represented according to their three-letter abbreviation and the number of their position in the protein.

Table S2. Interactions between CCMV protein and NDM-tamoxifen[a]

Pocket BMV-NDMT	Affinity energy (kCal/mol)	Hydrophobic interactions (#54) 88.5%		Hydrogen bonds (#7) 11.5%	
P1_CCMV_NDMT	-5.6	9	O/Val126, C2/Phe120, C12/Lys106, C19/Ala117, C20/Thr67, C21/Tyr159, C24/Ala123, C25/Ala125, C25/Leu124	0	-

P2_CCMV_NDMT	-5.6	7	N/Trp94, C3/Ala135, C9/Ile44, C10/Arg42, C18/Leu92, C22/Val133, C24/Val134	1	N/Leu95
P3_CCMV_NDMT	-5.0	7	C5/Leu95, C10/Asp132, C15/Trp94, C20/Asn129, C20/Val134, C21/Leu97, C25/Gly96	0	-
P4_CCMV_NDMT	-5.0	3	C5/Glu148, C13/Ala151, C22/Ala152	2	N/Leu74, N/Ser79
P5_CCMV_NDMT	-4.9	9	C9/Val122, C19/Glu111, C20/Phe142, C20/Thr110, C20/Val109, C22/Leu124, C23/Glu140, C25/Ala141, C25/Ile145	1	N/Lys143
P6_CCMV_NDMT	-4.9	7	C9/Phe182, C10/Thr187, C13/Ser80, C18/Asp183, C20/Ser185, C21/Phe186, C25/Asn76	0	-
P7_CCMV_NDMT	-4.8	7	N/Ile72, C6/Ala57, C9/Glu166, C16/Ser58, C20/Ala69, C21/Ala60, C21/Ile70, C24/Thr56, C25/Trp55	2	N/Ser73, N/Thr71
P8_CCMV_NDMT	-4.7	5	C6/Lys131, C16/Ala125, C16/Val126, C19/Asp128, C24/Val133	1	N/Asp132

[a]Affinity energies and interactions of drug-capsid protein obtained by molecular docking with Autodock Vina. Amino acids are represented according to their three-letter abbreviation and the number of their position in the protein.

NDM-tamoxifen characterization

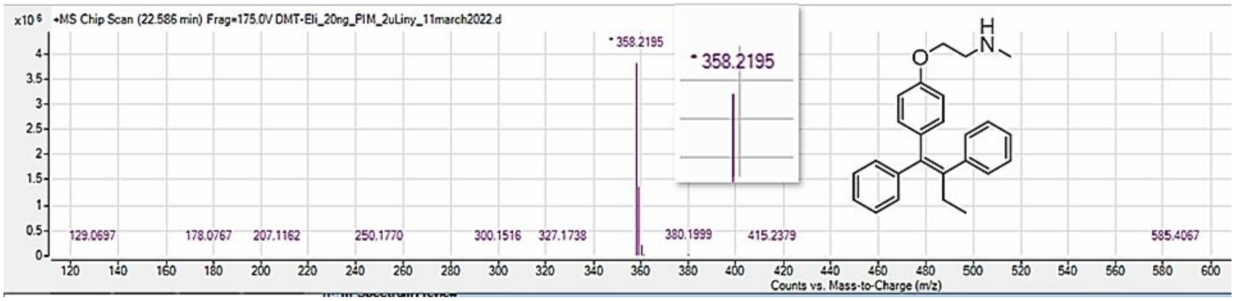

Figure S1. Extracted ion chromatogram: Ion chromatogram, highlighting the signal intensity corresponding to the NDM-tamoxifen molecule at 358.2195 m/z.

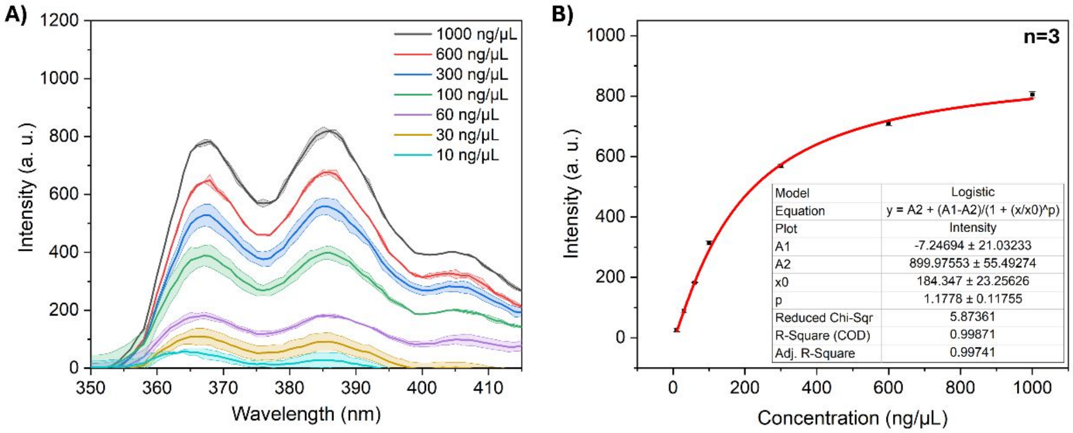

Figure S2. Emission spectra and calibration curves of the NDM-Tamoxifen fluorescence intensity as a function of concentration: A) Emission spectra of NDM-tamoxifen excited at a wavelength of 270 nm. B) Fluorescence intensity versus concentration curve of NDM-tamoxifen (n=3).

NDM-tamoxifen loading in BMV and CCMV

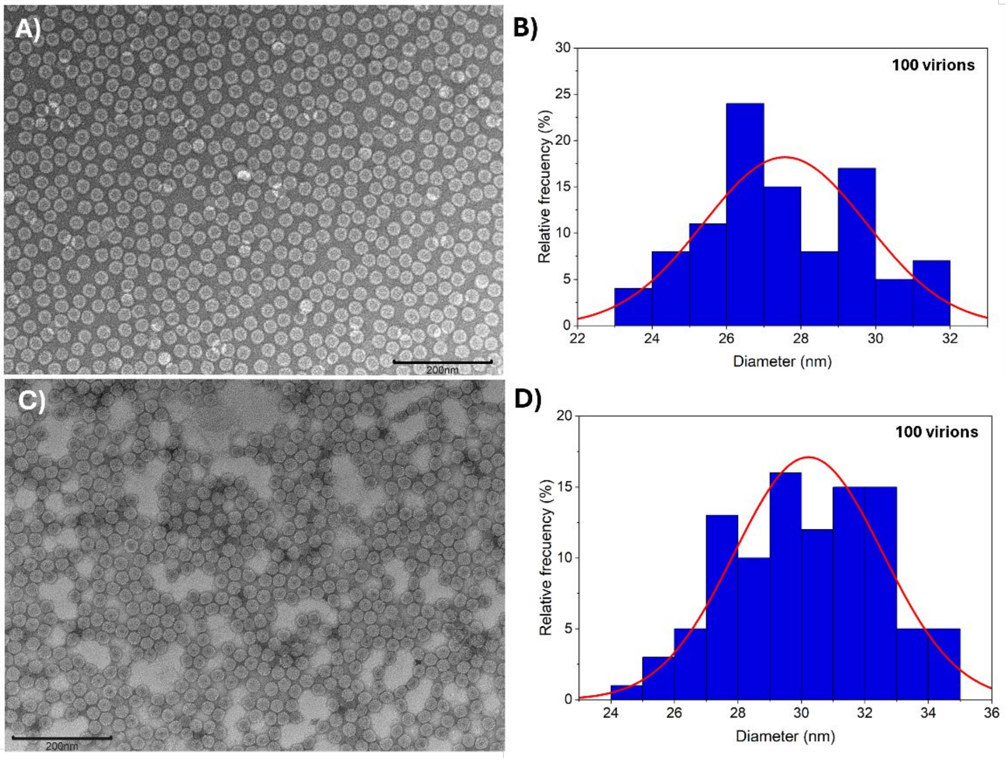

Figure S3. BMV and CCMV virion sizes: A) TEM micrographs of purified BMV viruses. B) Diameter distribution of BMV virions obtained by TEM counts. C) TEM micrographs of CCMVs obtained from TEM micrographs. D) Diameter distribution of BMV virions obtained by TEM counts. Magnifications 40 k×, scale 200 nm. The size distribution of VNPs was obtained by measure 100 virions.

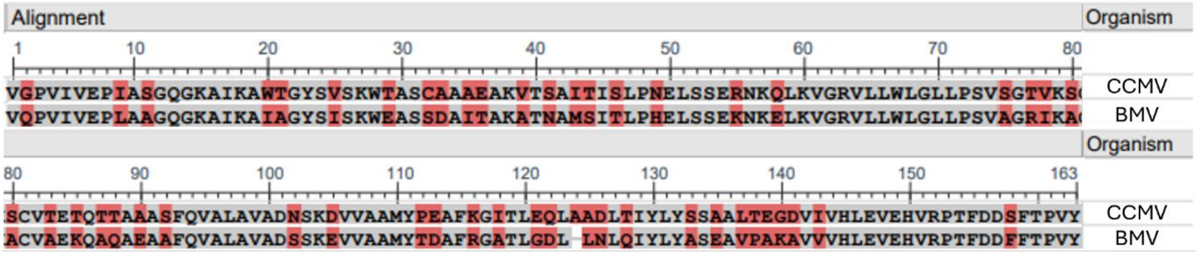

Figure S4. Alignment of the amino acid sequences of CCMV and BMV: Amino acids that differ between the two sequences are highlighted in red. Performed with NCBI Multiple Sequence Alignment Viewer, Version 1.25.3.

VNPs internalization in 4T1 cells

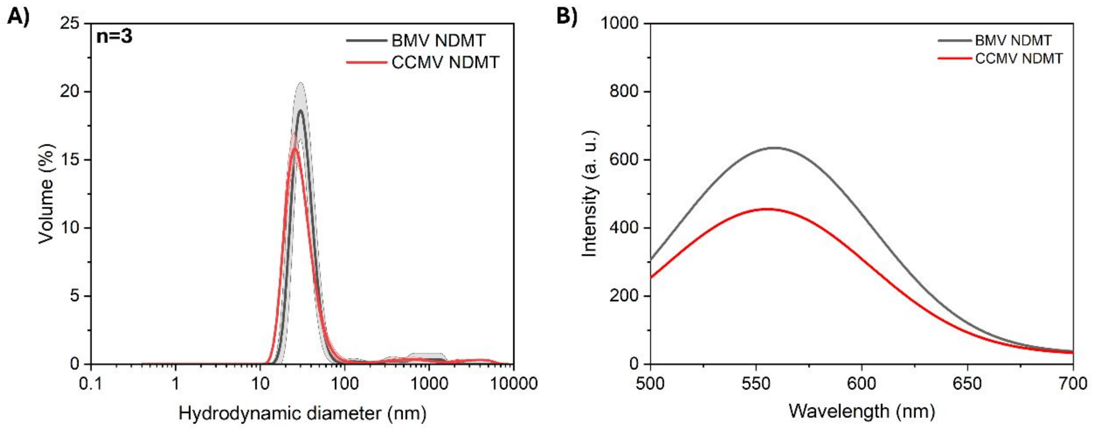

Figure S5. NanoOrange-marked VNPs: A) Hydrodynamic diameter of NanoOrange-marked VNPs, BMV-NDMT (gray), CCMV-NDMT (red) (n=3). B) Emission spectrum of NanoOrange-labeled VNPs, BMV-NDMT (gray), CCMV-NDMT (red).

Cell viability

Table S3. Virus and drug concentrations used to formulate VNPs at IC50 concentrations

**IC50**		**Virus component[a]**		**NDMT component[b]**		**VNP[c]**	
		**ng/µL (nM)**	**Particles per µL**	**ng/µL (nM)**	**Molecules per µL**	**ng/µL (nM)**	**Particles per µL**
**MDA-MB-231**	**BMV-NDMT**	31.80 (6.91)	4.16×10^{9}	4.95 (13,846.15)	8.34×10^{12}	36.75 (7.98)	4.16×10^{9}
	**CCMV-NDMT**	102.54 (22.29)	1.34×10^{10}	7.8 (21,828.18)	1.31×10^{13}	110.34 (23.98)	1.34×10^{10}
**4T1**	**BMV-NDMT**	111.59 (24.25)	1.46×10^{10}	17.37 (48,587.41)	2.93×10^{13}	128.96 (28.03)	1.46×10^{10}
	**CCMV-NDMT**	217.19 (47.21)	2.84×10^{10}	16.52 (46,209.79)	2.78×10^{13}	233.71 (50.80)	2.84×10^{10}

[a]virus concentration in the nanoformulation. [b]NDMT concentration in the nanoformulation. [c]VNP concentration at IC50.

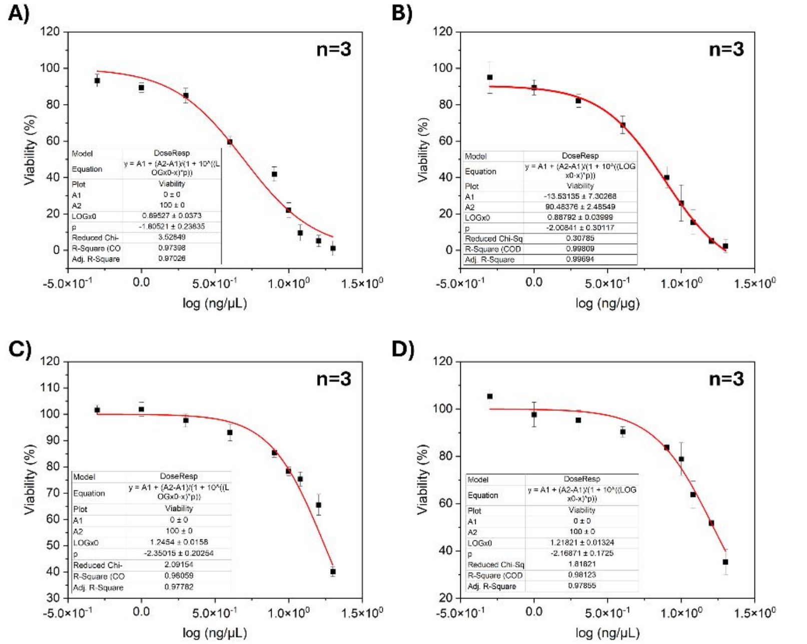

Figure S6. Dose/response determination in MDA-MB-231 and 4T1 cells: A) Dose/response effect of BMV-NDMT in MDA-MB-231 cells. B) Dose/response effect of CCMV- NDMT in MDA-MB-231 cells. C) Dose/response effect of BMV-NDMT in 4T1 cells. D) Dose/response effect of CCMV- NDMT in 4T1 cells. Points represent the average viability, and error bars represent the standard deviation (SD), n=3.

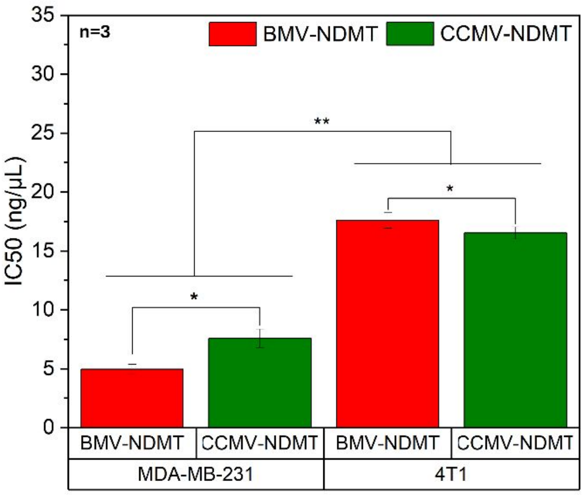

Figure S7. Comparison of the IC_{50} results: The effect of BMV-NDMT treatment is shown in red, and the effect of CCMV-NDMT treatment is shown in green in both cell lines (n=3). Bars represent the IC_{50} and error bars represent the standard deviation (SD). Statistical analyses One-way ANOVA, Tukey test, *P < 0.05, **P < 0.005.

Murine model:

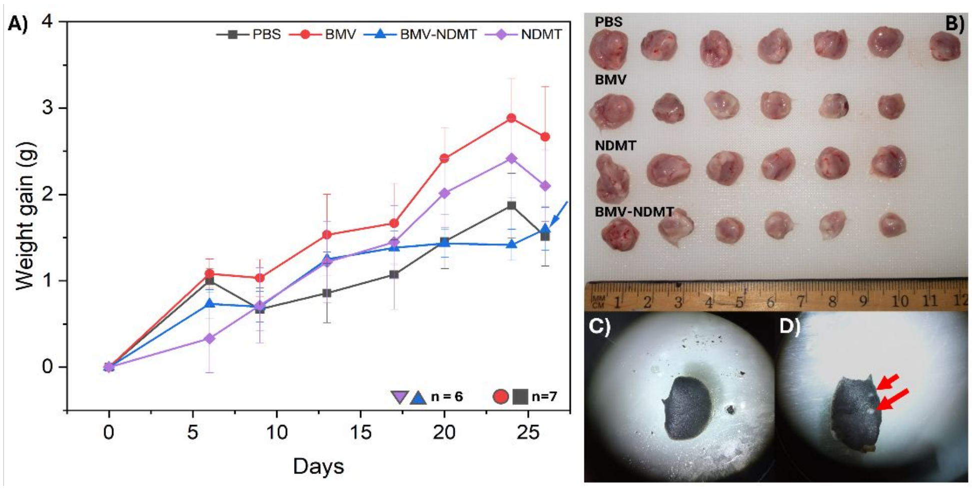

Figure S8. Weight of the mice. Photographs of the removed tumors and lung lobes: A) Weight gain of the mice (n = 6 for NDMT and BMV, n=7 for PBS and BMV-NDMT). B) Photograph of removed breast cancer tumors. C) Photograph of a lung lobe without metastatic tumors. D) Photograph of a lung lobe with metastatic breast tumors (red arrows).
